# Acteoside From *Ligustrum robustum* (Roxb.) Blume Ameliorates Lipid Metabolism and Synthesis in a HepG2 Cell Model of Lipid Accumulation

**DOI:** 10.3389/fphar.2019.00602

**Published:** 2019-05-24

**Authors:** Le Sun, Fan Yu, Fan Yi, Lijia Xu, Baoping Jiang, Liang Le, Peigen Xiao

**Affiliations:** ^1^Institute of Medicinal Plant Development, Chinese Academy of Medical Sciences, Peking Union Medical College, Beijing, China; ^2^Key Laboratory of Bioactive Substances and Resources Utilization of Chinese Herbal Medicine, Ministry of Education, Beijing, China; ^3^Key Laboratory of Cosmetics, China National Light Industry, Beijing Technology and Business University, Beijing, China

**Keywords:** acteoside, *in silico* screening, RNA-seq, lipid metabolism and synthesis, *Ligustrum robustum* (Roxb.) Blume

## Abstract

We aimed to ascertain the mechanism underlying the effects of acteoside (ACT) from *Ligustrum robustum* (Roxb.) Blume (Oleaceae) on lipid metabolism and synthesis. ACT, a water-soluble phenylpropanoid glycoside, is the most abundant and major active component of *L. robustum*; the leaves of *L. robustum*, known as kudingcha (bitter tea), have long been used in China as an herbal tea for weight loss. Recently, based on previous studies, our team reached a preliminary conclusion that phenylpropanoid glycosides from *L. robustum* most likely contribute substantially to reducing lipid levels, but the mechanism remains unclear. Here, we conducted an *in silico* screen of currently known phenylethanoid glycosides from *L. robustum* and attempted to explore the hypolipidemic mechanism of ACT, the representative component of phenylethanoid glycosides in *L. robustum*, using RNA-seq technology, quantitative real-time PCR (qPCR) and Western blotting. First, the screening results for six compounds were docked with 15 human protein targets, and 3 of 15 protein targets were related to cardiovascular diseases. Based on previous experimental data and docking results, we selected ACT, which exerted positive effects, for further study. We generated a lipid accumulation model using HepG2 cells treated with a high concentration of oleic acid and then extracted RNA from cells treated for 24 h with 50 μmol/L ACT. Subsequently, we performed a transcriptomic analysis of the RNA-seq results, which revealed a large number of differentially expressed genes. Finally, we randomly selected some genes and proteins for further validation using qPCR and Western blotting; the results agreed with the RNA-seq data and confirmed their reliability. In conclusion, our experiments proved that ACT from *L. robustum* alters lipid metabolism and synthesis by regulating the expression of multiple genes, including *Scarb1*, *Scarb2*, *Srebf1*,* Dhcr7*, *Acat2*, *Hmgcr*, *Fdft1*, and *Lss*, which are involved several pathways, such as the glycolytic, AMPK, and fatty acid degradation pathways.

## Introduction

As the global economy has increased and altered people’s lifestyles, cardio-cerebrovascular diseases have become the top cause of death in humans worldwide, and the mortality rate of these diseases has exceeded that of cancer (WHO, 2013). According to a substantial number of studies, hyperlipidemia is a primary risk factor for coronary heart disease, atherosclerotic cardiovascular disease, stroke, myocardial infarction, cerebrovascular accidents, sudden cardiac death, and so on. Hence, effective clinical treatments must be developed and administered to lower lipid levels and improve dyslipidemia, changes that will subsequently decrease the morbidity and mortality of cardio-cerebrovascular diseases (Kopin and Lowenstein, 2010; Zoungas et al., 2014). Recently, research on hypolipidemic therapies designed to curtail cardiovascular events has mainly focused on chemical drugs, such as statins, as a primary and effective therapy for people with high plasma cholesterol levels and increased cardiovascular risk; these drugs have been typically prescribed as lifelong therapies (Zoungas et al., 2014). Although some researchers believe that the benefits of statin therapy far overweigh its side effects, the use of highly potent statins causes some unavoidable adverse effects, such as chronic or acute kidney injury, skeletal myotoxicity, and diabetes (Graham et al., 2004; Sattar et al., 2010; Dormuth et al., 2013). Therefore, the search for new compounds derived from natural products has become a research hotspot.

Currently, with the changing definition of health, people are increasingly preferring complementary and alternative medicines to prevent chronic diseases, such as exercise, a balanced diet, and tea consumption rather than clinical treatments. In fact, daily tea consumption is prevalent and is usually regarded as an effective method to prevent and ameliorate chronic metabolic diseases (e.g., hyperlipidemia and obesity) (Huang et al., 2009). Kuding tea [(leaves of *Ligustrum robustum* (Roxb.) Blume (Oleaceae)] has a long history of being utilized as a drink in China, and *L. robustum* is one of the major botanicals cultivated in southwestern areas. In China, kuding tea has traditionally been used as an antiobesity agent.

Water-soluble total phenylpropanoid glycosides are the characteristic constituents of *L. robustum*, and the total contents of five characteristic phenylethanoid glycosides are 144 to 158 mg/g (Li et al., 2012). As shown in our previous study, total phenylpropanoid glycosides of *L. robustum* (CNTG) exert significant hypolipidemic effects on high-fat diet-induced obesity in C57BL/6J mice, and the efficacy might be related to the upregulation of leptin gene expression (Yang et al., 2015). In our subsequent study, CNTG promoted liver kinase B1 (LKB1) phosphorylation to activate adenosine 5′-monophosphate (AMP)-activated protein kinase (AMPK) and then restrained sterol regulatory element-binding protein (SREBP-1c) activity to reduce the expression of key downstream enzymes, including fatty acid and TG synthases (Sun et al., 2017; Yang et al., 2018). As mentioned above, CNTG probably plays a primary role in the hypolipidemic effects of *L. robustum*.

Acteoside (ACT), also known as kusagin or verbascoside, is the most abundant and major active component of CNTG, and the content in old leaves is 20 to 50 mg/g (Li et al., 2012). It has been proven to possess several extensive biological activities, such as antioxidant, anti-inflammatory, antitumor, and neuroprotective effects (Gan et al., 2018; Li et al., 2018). Recently, we performed an *in vitro* evaluation of the main compounds in *L. robustum* and discovered that lipid accumulation in HepG2 cells treated with oleic acid was significantly reduced by different concentrations of ACT, ligupurpuroside A, ligupurpuroside C, and ligupurpuroside D (Yang et al., 2018). ACT inhibits the activity of pancreatic lipase and is associated with reduced fatty acid absorption (Wu et al., 2014). However, the potential mechanism underlying the hypolipidemic effects of ACT remains unclear.

Transcriptomics, one of the most important aspects of functional genomics, is applied to study the regulation of transcription and the overall transcription of all genes in the cells. Based on the distinct gene expression profiles between the two groups, we could identify key genes regulated by drugs. This article was devoted to identifying the potential signal transduction pathway and preliminary protein targets. First, we performed an *in silico* investigation using pharmacophore-based molecule docking experiments to predict potential compounds in CNTG and targets that might be involved in hyperlipidemia. Afterward, we analyzed the transcriptome of the ACT-treated HepG2 cell lipid accumulation model to explore its potential hypolipidemic mechanism. Finally, we performed qPCR analysis of HepG2 cells to further validate the RNA-seq data. In summary, this article aimed to provide relevant mechanistic evidence for the hypolipidemic effects of ACT from *L. robustum*.

## Materials and Methods

### *In Silico* Prediction

Following a literature review, we selected 13 phenylglycoside components from *L. robustum* (**Supplementary File S1**). Biovia Discovery Studio (DS 3.5) was used to calculate the absorption, distribution, metabolism, and excretion (ADME)-related pharmacokinetic properties of these 13 compounds, including their bioavailability (OB), drug-likeness (DL), and distribution across the blood–brain barrier (BBB). OB values ≥ 30% and DL ≥ 0.18 were used as ADME screening criteria for candidate compounds, and we ultimately identified six compounds. All six phenylglycosides were subjected to target fishing with the PharmaDB database in DS. The RIGID molecular overlapping algorithm model was utilized to calculate the molecular features and ensure that they were all collected (Meslamani et al., 2011; Meslamani et al., 2012). All calculations were performed on a DELL Precision T5610 workstation with 8.00 GB of system memory, an Inter Xeon CPU E5-2609 v2@ 2.50 GHz processor, and a Windows 7 professional system.

Target proteins and signaling pathway information were included in the Protein Data Bank (PDB) database, and the Database for Annotation, Visualization, and Integrated Discovery (DAVID, v6.8, https://david.ncifcrf.gov/) was used for the gene ontology (GO) analysis and Kyoto Encyclopedia of Genes and Genomes (KEGG) pathway enrichment analysis. Target-related pathways and disease information were obtained from the TTD and DrugBank databases (Zhu et al., 2012). A compound-target-disease interaction map was constructed using Cytoscape 3.0 software (Smoot et al., 2011) by analyzing the hit targets, associated proteins and diseases, and interactions between these parameters.

### Cell Culture and Treatment

HepG2 human hepatoma cells (Institute of Basic Medical Sciences, Chinese Academy of Medical Sciences) were grown in Dulbecco’s modified Eagle’s medium (DMEM, HyClone) supplemented with 10% fetal bovine serum (FBS, Gibco) and 1% penicillin/streptomycin (HyClone) in a humidified 5% CO_2_ atmosphere at 37°C. ACT (CAS: 61276-17-3) was purchased from Sigma, and its purity was ≥ 99%.

Cells were cultured in 10-cm^2^ culture dishes at a density of 1 × 10^5^ cells per milliliter. After the cells had completely attached, they were divided into three groups, the control, model, and ACT groups, with two replicates per group. The medium of the three groups was replaced with normal DMEM, DMEM containing 100 μmol/L oleic acid, or DMEM containing 100 μmol/L oleic acid and 50 μmol/L ACT, and the cells were cultured for 24 h.

### RNA Isolation and Sequencing 

Total RNA was extracted from the three groups of cells using the RNAsimple Total RNA Kit. RNA purity was assessed using a NanoPhotometer® spectrophotometer (IMPLEN, Westlake Village, CA). RNA concentrations were measured using a Qubit® RNA Assay Kit with a Qubit® 2.0 Fluorometer (Life Technologies, Carlsbad, CA). RNA integrity was assessed using the RNA Nano 6000 Assay Kit and the Bioanalyzer 2100 system (Agilent Technologies, Santa Clara, CA).

Sequencing libraries were generated using the NEBNext® Ultra™ RNA Library Prep Kit for Illumina® (NEB, USA) according to the manufacturer’s recommendations, and index codes were added to attribute sequences to each sample. After cluster generation, the library preparations were sequenced using the Illumina HiSeq platform, and 125/150 bp paired-end reads were generated.

### RNA-seq Data and Pathway Analysis

Clean reads were obtained by removing reads containing adapter or poly-N sequences and low-quality reads from the raw data. At the same time, the Q20, Q30, and GC contents of the clean data were calculated. Reference genome and gene model annotation files were downloaded directly from the genome website. The index of the reference genome was built using Bowtie v2.2.3, and paired-end clean reads were aligned to the reference genome using TopHat v2.0.12 (Trapnell et al., 2009).

Differentially expressed genes between the two conditions/groups (two biological replicates per condition) were identified using the DESeq R package (1.18.0) (Anders and Huber, 2010). The resulting P values were adjusted using the Benjamini and Hochberg approach for controlling the false discovery rate. Genes with an adjusted P-value < 0.05 identified using DESeq were assigned as differentially expressed. GO terms with corrected P values less than 0.05 were considered significantly enriched by the differentially expressed genes. We used KOBAS software to test the statistical significance of the enrichment of differentially expressed genes in KEGG pathways (Kanehisa et al., 2008; Young et al., 2010).

### Validation by Quantitative Real-Time PCR

Cells were cultured as described in the section Cell Culture and Treatment.

Cells were cultured in 6-well plates at a density of 1 × 10^5^ cells per milliliter. After the cells had completely attached, they were divided into three groups, the control, model, and ACT groups, with six replicates per group. The medium of each group was replaced with normal DMEM, DMEM containing 100 μmol/L oleic acid, or DMEM containing 100 μmol/L oleic acid and 50 μmol/L ACT, and the cells were cultured for 24 h.

Total RNA was extracted using the RNAsimple Total RNA Kit. cDNA was synthesized using the PrimeScript RT Reagent Kit according to the manufacturer’s protocol. PCR was performed using the SYBR PrimeScript Ex TaqII Kit with the following reaction conditions: 40 cycles of 95°C for 30 s, 60°C for 30 s, and 72°C for 30 s. All PCR primers were designed using Primer Premier 5.0 and Oligo 6 Demo Software (**Table 1**) and were synthesized by BioSino Biotechnology and Science. Target gene expression was normalized to the expression of the *Gapdh* gene, and relative expression was calculated using the 2^−ΔΔCt^ method with REST ^©^2009 software.

**Table 1:** Quantitative real-time PCR primer sequences.

Gene	Primers (5’-3’)	
*Dhcr7*	F: GCTCATGTACTCCGTGACGA	R: GGTCACAAGCCATGATGAAG
*Acat2*	F: CCGGAAGATGTGTCTGAGGT	R: GCTCCATGCTGGAACAGAGT
*Hmgcr*	F: GCCTGGCTCGAAACATCTGAA	R: CTGACCTGGACTGGAAACGGATA
*Lss*	F: ATATGCGCTCCTCAACCTGT	R: GATGCTGATGCTCTTGGTGA
*Fdft1*	F: GGAAGACCAGCAAGGAGGAA	R: ACTGCACGGCCAAGTCAATA
*Scarb1*	F: GGCTGAGCAAGGTTGACTTC	R: AGAACTCCAGCGAGGACTCA
*Scarb2*	F: AGAAGGCATGCACCCAAATC	R: TTTGGAACCTCTTGGCTGCT
*Srebf1*	F: GCACTTTTTGACACGTTTCTTC	R: CTGTACAGGCTCTCCTGTGG
*Cpt1a*	F: AGCGACTGGTGGGAGGAGTA	R: GCTCTTGCTGCCTGAATGTG
*Gapdh*	F: AGGTCGGAGTCAACGGATTTG	R: GTGATGGCATGGACTGTGGT

### Western Blotting Analysis

Protein samples were prepared from the cells according to the manufacturer’s instructions (ComWin Biotech, China) and quantified using the bicinchoninic acid (BCA) method. Ten micrograms of each protein sample was separated by SDS-polyacrylamide gel electrophoresis and then transferred onto CN membranes. Membranes were subsequently blocked with 5% skim milk and incubated with primary antibodies against SREBF1 (1:4000; Abcam, UK), LSS (1:4000; Abcam), HMGCR (1:4000; Abcam), FDFT1 (1:1000; Santa Cruz Biotech, Santa Cruz, CA), CPT1a (1:1000; Santa Cruz Biotech), SCARB2 (1:1000; Santa Cruz Biotech), and β-actin (1:4000, Beijing ComWin Biotech Co., Ltd.) overnight at 4°C. The membranes were then washed three times with TBS-T buffer and incubated with a horseradish peroxidase (HRP)-conjugated secondary antibody (1:4,000, Beijing ComWin Biotech Co., Ltd.) for 1 h at room temperature. The membranes were washed three times, followed by immunodetection using an enhanced chemiluminescence kit (Beijing ComWin Biotech Co., Ltd.).

## Results

### Protein-Ligand Docking

**Figure 1:**
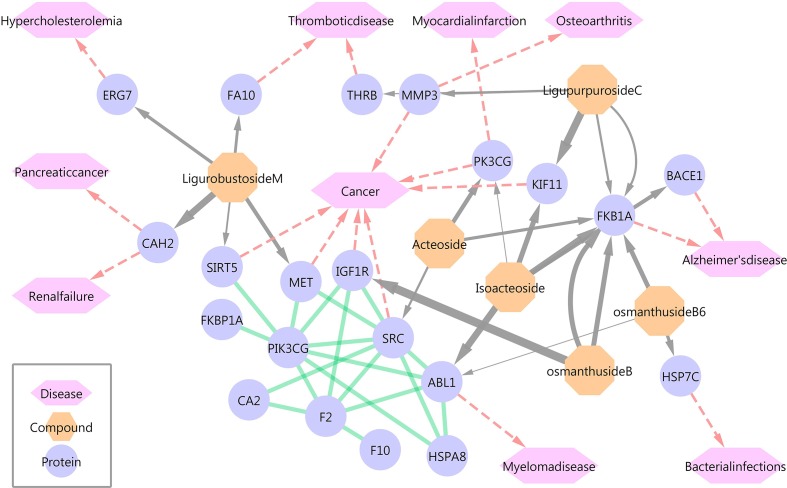
Overview of the compound-target-disease relationships. Gray lines represent compound-protein relationships, and the thickness is proportional to the fitscore value. Red lines represent protein-disease relationships. Green lines represent protein interactions.

Six of the 13 phenylpropanoid glycosides were docked with 68 protein targets, of which 15 human protein targets were verified by the TTD and DrugBank databases for further analysis. **Figure 1** and **Supplementary File S2** provide an overview of the compound-target-disease relationships.

Traditional analyses of the efficacy of phenylethanoid glycosides have focused on antitumor, antibacterial, and neuroprotective effects, among others, but few studies have examined their effects on cardiovascular diseases. Therefore, more tumor-related and Alzheimer’s disease-related targets were identified in this screen. In addition, 3 of 15 protein targets were related to cardiovascular diseases, such as hypercholesterolemia, thrombus, and myocardial infarction. ACT and isoacteoside docked with phosphatidylinositol-4,5-bisphosphate 3-kinase, catalytic subunit gamma (PIK3CG), which has an important relationship with myocardial infarction. Ligurobustoside M bound to LSS, which plays a role in the steroid biosynthesis pathway and may have a relationship with hypercholesterolemia.

### RNA-seq Analysis of Differentially Expressed Genes

**Figure 2:**
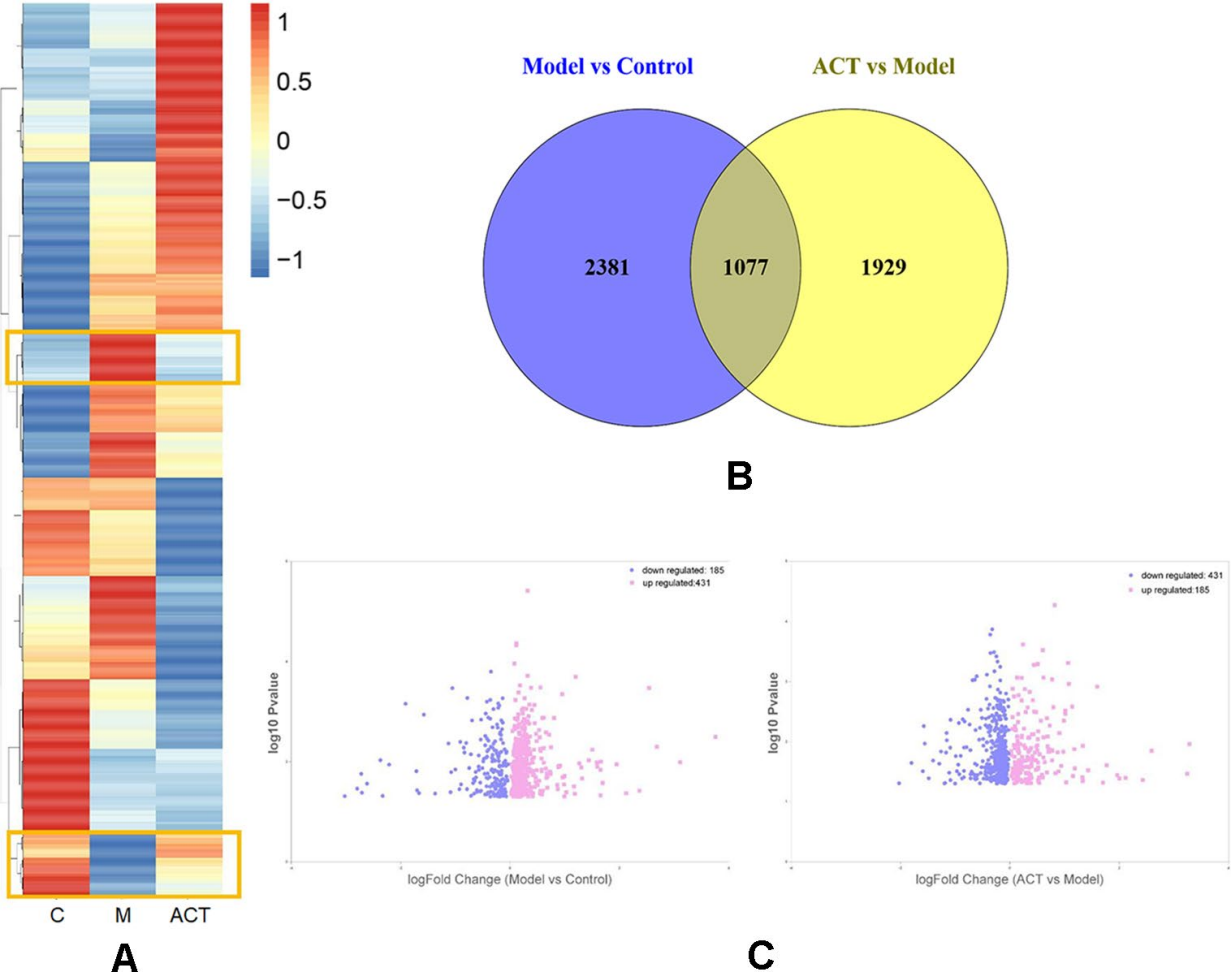
Comparison of gene expression levels in HepG2 cells from the model and control groups and from the ACT and model groups. **(A)** Heatmap showing the results of the correlation analysis of key metabolites among the control, model and ACT groups. **(B)** Differentially expressed genes between the model and control groups and between the ACT and model groups are shown in the Venn diagram. **(C)** In the volcano plot, the horizontal axis represents the log2-fold change; the more a point deviates from the center, the wider the disparity. The vertical axis represents the -log10 P-value; a higher value indicates a more significant difference between the model and control groups and between the ACT and model groups.

A hierarchical clustering analysis was performed according to the FPKM values of differentially expressed genes identified in cells cultured under different experimental conditions, which were normalized and clustered according to the log10 (FPKM+1) value. The expression patterns of these genes are presented as squares in the heatmap (**Figure 2A**), with normalized values displayed based on a specific color gradient: red indicates genes expressed at high levels, and blue indicates genes expressed at low levels. As the color transitions from red to blue, the log_10_ (FPKM+1) value decreases. The area marked with a yellow frame in **Figure 2A** indicates genes with lower (higher) expression in the normal group that were upregulated (downregulated) in the model group treated with oleic acid but downregulated (upregulated) in response to ACT treatment. Based on these data, the marked genes probably influence lipid metabolism in HepG2 cells exposed to ACT.

Afterward, according to the condition of P_adj_ < 0.05, we identified significantly differentially expressed genes in the control group compared with the model group and in the model group compared with the ACT group (Oliveros, 2007). As shown in **Figure 2B**, 3,458 differentially expressed genes were identified in the model group compared with the normal group, and 3,006 differentially expressed genes were identified in the ACT group compared with the model group. Moreover, 1,077 differentially expressed genes overlapped between the model and control groups and between the ACT and model groups, indicating that these overlapping genes were affected by both oleic acid and ACT.

However, we had not yet determined whether the expression levels of these 1,077 genes conformed to the trends described in **Figure 2A**. Ultimately, 616 genes showed a consistent trend: genes with low (high) expression levels in the normal group were upregulated (downregulated) in the model group after treatment with oleic acid and downregulated (upregulated) after treatment with ACT, as depicted in **Figure 2C** and **Supplementary File S3**. Four hundred thirty-one upregulated genes and 185 downregulated genes were identified in the model group compared to the control group, and they presented opposite trends in the ACT group compared to the model group.

### GO and KEGG Pathway Analyses

**Figure 3:**
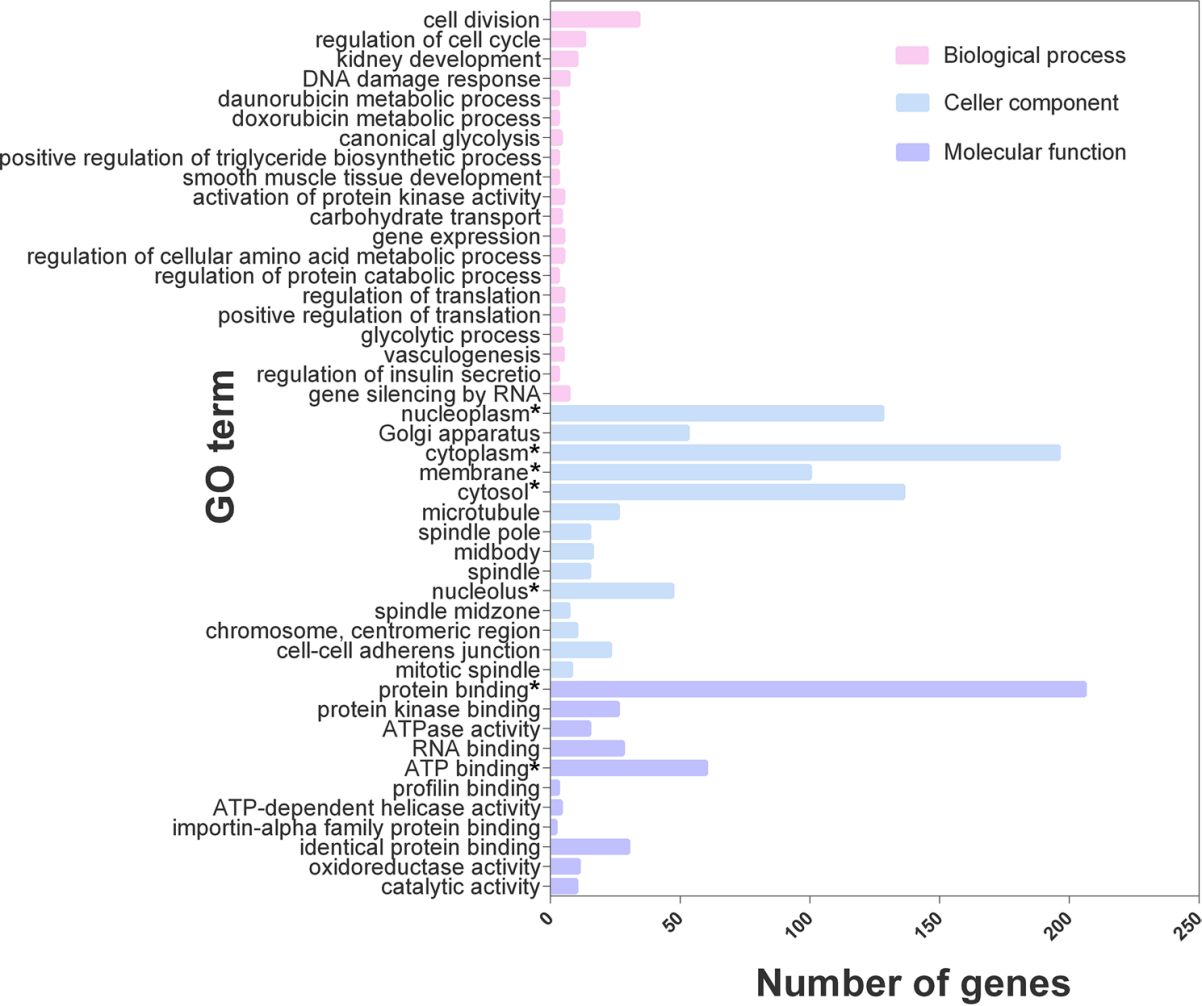
GO enrichment analysis of the top 45 terms for 616 genes. *P value < 0.05.

Based on the GO functional annotation and enrichment analysis, 548 of the 616 differentially expressed genes were enriched in 155 GO terms, and 45 GO terms were related to lipid metabolism and synthesis (**Figure 3**), including the metabolism of doxorubicin, positive regulation of triglyceride biosynthesis, glycolytic process, carbohydrate transportation, regulation of cellular amino acid metabolic process, and regulation of insulin secretion. The differentially expressed genes were mainly involved in the biosynthesis and metabolism of cholesterol and triglycerides, glycolytic metabolism, and insulin secretion processes.

**Figure 4:**
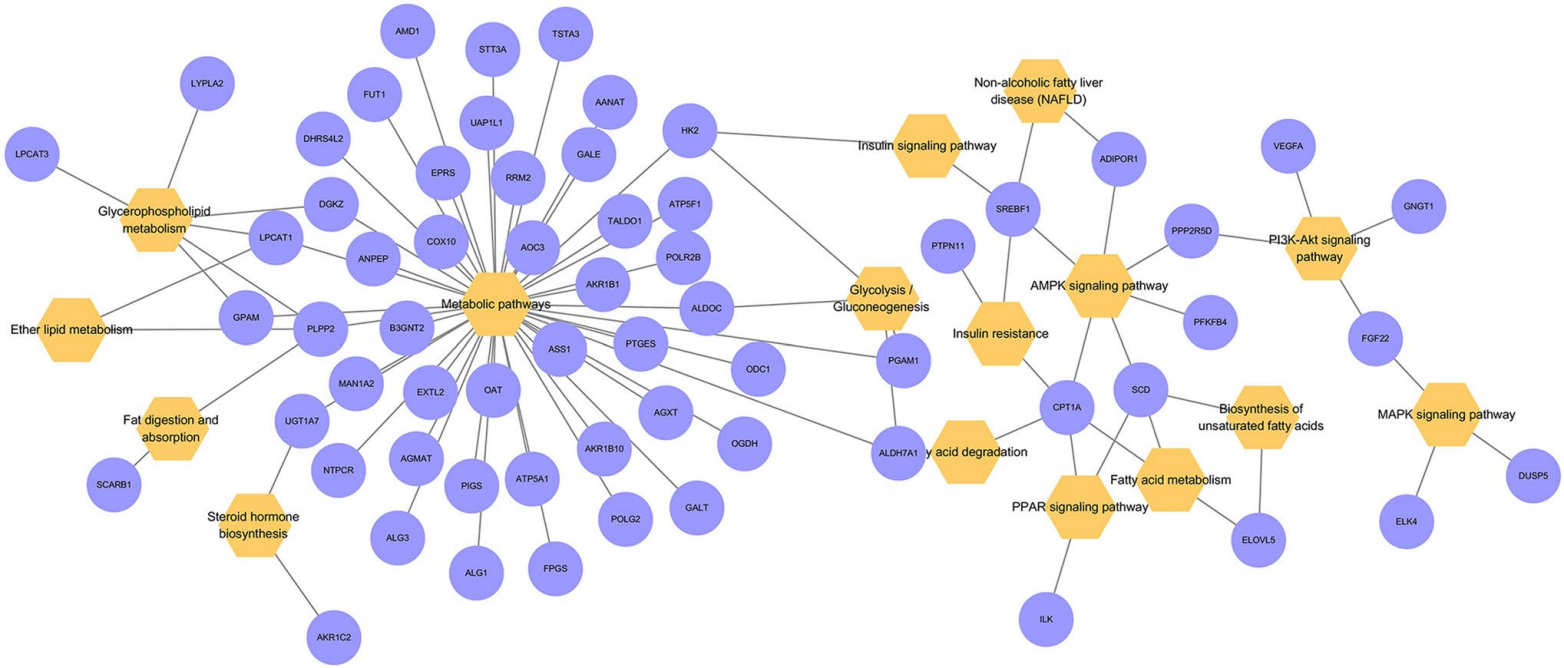
Bubble diagram of the KEGG pathway enrichment analysis. Orange represents enriched KEGG pathway terms. Purple represents differentially expressed genes in related pathways.

A bubble diagram (**Figure 4**) was used to visualize the results of the KEGG pathway enrichment analysis, which involved lipid metabolism in an intuitive and explicit manner. The 616 differentially expressed genes were matched to several lipid metabolism pathways, including the metabolic, glycolysis, AMPK, fatty acid degradation, and fatty acid metabolism pathways.

### qPCR Validation

**Figure 5:**
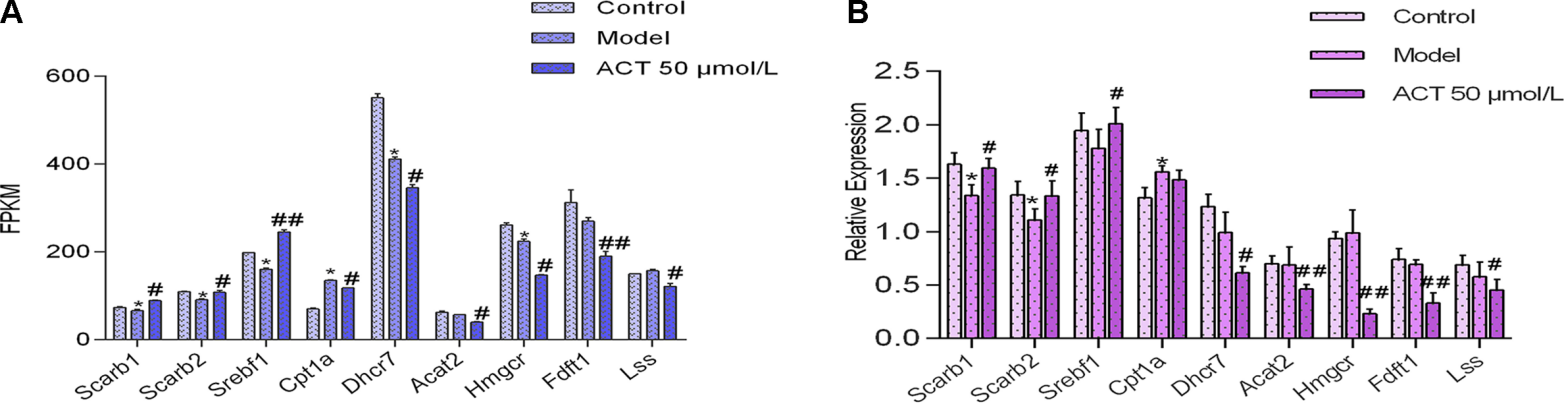
The mRNA expression levels of genes involved in lipid metabolism in HepG2 cells. Data are presented as the means ± SEMs. *P < 0.05 compared with the control group; #*P* < 0.05 and ##*P* < 0.01 compared with the model group (n = 3). **(A)** FPKM values for *Scarb1*, *Scarb2*, *Srebf1*, *Cpt1a*, *Dhcr7*, *Acat2*, *Hmgcr*, *Fdft1*, and *Lss* in the RNA-seq data. **(B)** *Scarb1*, *Scarb2*, *Srebf1*, *Cpt1a*, *Dhcr7*, *Acat2*, *Hmgcr*, *Fdft1*, and *Lss* mRNA expression levels measured by qPCR.

The gene expression levels of key enzymes involved in lipid metabolism were assessed in HepG2 cells to validate the aforementioned metabolic changes and virtual target predictions. First, based on the virtual screening and RNA-seq results, we randomly selected several genes for verification using qPCR, including *Scarb1*, *Scarb2*, *Srebf1*, and *Cpt1a*. In addition, we randomly selected several genes for verification by qPCR, including *Dhcr7*, *Acat2*, *Hmgcr*, *Fdft1*, and *Lss*, to explore whether ACT directly regulates lipid synthesis and metabolism in HepG2 cells by comparing the ACT and model groups. The expression of these genes was normalized to the *Gapdh* mRNA expression. The RNA-seq and qPCR results are shown in **Figures 5A and B**, respectively. Compared with the control group, the model group showed increased levels of *Cpt1a* mRNA (*P* < 0.05) and decreased expression of *Scarb1* and *Scarb2* mRNA (*P* < 0.05); compared with the model group, the ACT group showed a significant decrease in *Acat2*, *Dhcr7*, *Fdft1*, *Lss*, and *Hmgcr* mRNA expression (*P* < 0.05) and an increase in *Scarb1*, *Scarb2*, and *Srebf1* mRNA expression (*P* < 0.05).

### Effect of ACT on Related Protein Levels

**Figure 6:**
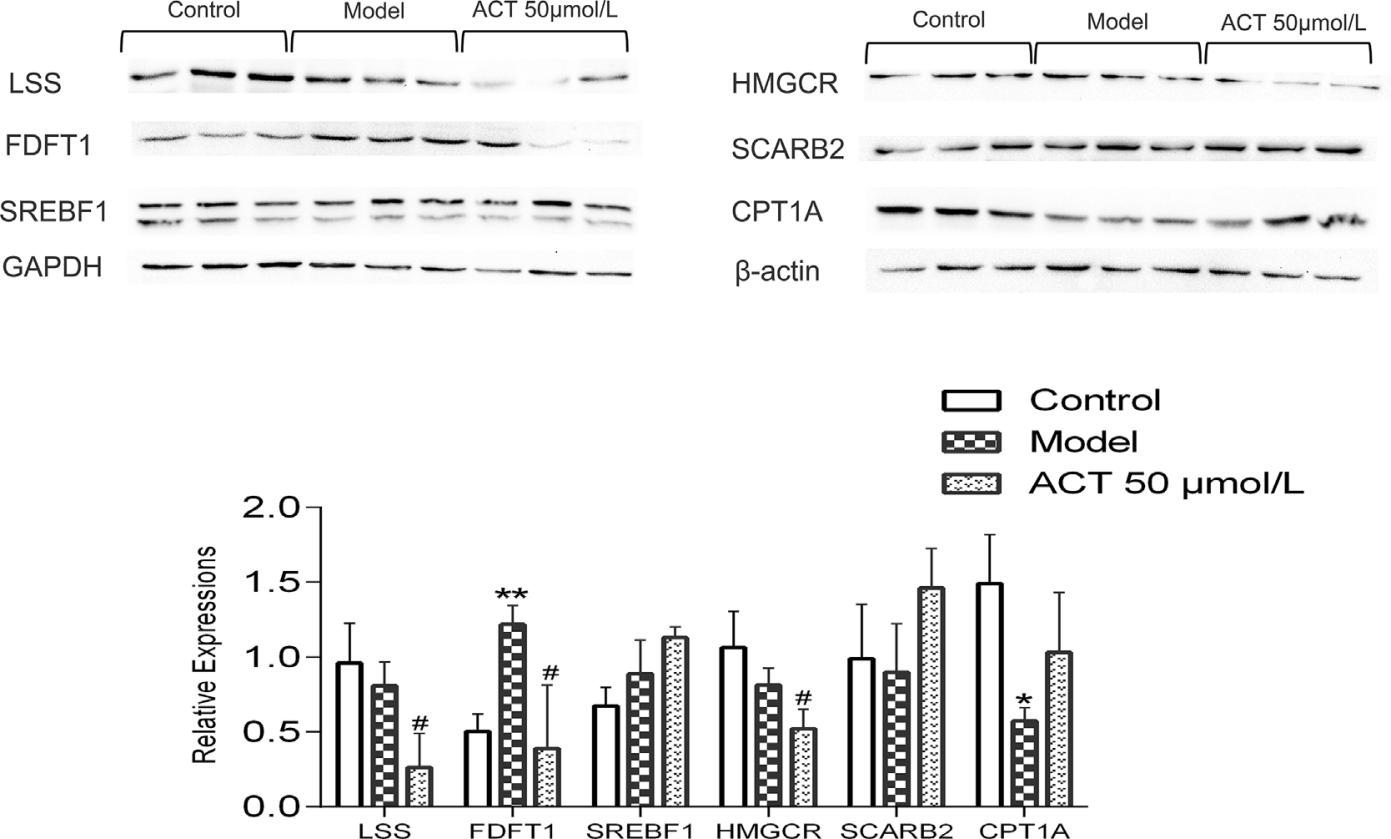
The expression levels of SREBF1, LSS, HMGCR, FDFT1, CPT1A, and SCARB2 in HepG2 cells. Data are presented as the means ± SEMs. *P < 0.05 and **P < 0.01 compared with the control group; #*P* < 0.05 compared with the model group (n = 3).

SREBF1, LSS, HMGCR, FDFT1, CPT1A, and SCARB2 protein levels were determined by Western blotting to further confirm the potential mechanism of ACT. The expression levels of these proteins were normalized to the GAPDH and β-actin protein expression levels. The gray value was calculated by ImageJ software. As shown in **Figure 6**, compared with the control group, the model group showed increased levels of FDFT1 (*P* < 0.05) and decreased expression of CPT1A (*P* < 0.05); compared with the model group, the ACT group showed significantly decreased protein expression of LSS, HMGCR, and FDFT1 (*P* < *0.05*). However, the protein levels of SREBF1, SCARB2, and CPT1A were not significantly different after treatment with ACT.

## Discussion and Conclusions

Most of the existing research focuses on other pharmacological activities of ACT, such as antitumor, antibacterial, and neuroprotective effects, and few studies have examined its lipid-lowering effects. Our previous work showed that phenylethanoid glycosides from *L. robustum* regulate lipid metabolism and synthesis, but the mechanism was unclear. This study provides reliable data for further investigations of the hypolipidemic effect and mechanism of phenylethanoid glycosides from *L. robustum*.

The results of the *in silico* screen revealed six compounds that docked with 15 human target proteins, which were linked to hyperlipidemia, Alzheimer’s disease, cancer, and antibacterial activity. Among them, PIK3CG was captured by ACT and cisacteoside, and LSS was captured by ligurobustoside M. Recently, PIK3CG inhibitors were shown to significantly improve diabetes-induced cardiomyopathy, suggesting that ACT maybe a potential drug for the treatment of myocardial infarction (Maffei et al., 2017).

This article investigated the possible mechanism of ACT in improving lipid accumulation in HepG2 cells by analyzing the transcriptome to draw additional conclusions. By comparing the number of differentially expressed genes between the model and control groups and between the ACT and model groups, 1077 differentially expressed genes that overlapped between two groups were identified, indicating that the expression of these genes was affected by both oleic acid and ACT. However, only 616 genes conformed to the filter criteria. Among these genes, 431 were upregulated, and 185 were downregulated in the model group compared with the control group. The expression of these genes displayed the opposite result following treatment with ACT, namely, 185 genes were upregulated and 431 genes were downregulated in the ACT group compared with the control group. Further analyses of these differentially expressed genes by GO and KEGG enrichment analyses showed that a large proportion were involved in multiple complex processes, such as biological processes, molecular function, and cellular components, and were enriched in multiple pathways related to lipid metabolism. Based on these results, ACT potentially regulates lipid metabolism in the HepG2 model of lipid accumulation at multiple levels and through multiple pathways.

Finally, according to the virtual screening results and RNA-seq data combined with GO functional annotation and KEGG pathway enrichment analysis, some of the differentially expressed genes were randomly selected for verification by qPCR and Western blotting analysis, including *Scarb1*, *Scarb2*, *Srebf1*, *Cpt1a*, *Dhcr7*, *Acat2*, *Hmgcr*, *Fdft1*, and *Lss*. The verification accuracy of qPCR reached 80%, and the accuracy of Western blotting was approximately 50%. These data prove to some extent the reliability of RNA-seq analysis.

The functions of these genes and proteins were annotated by referring to the UniProt database and literature. DHCR7, LSS, and FDFT1 are important targets in the steroid biosynthetic pathway and are involved in cholesterol biosynthesis. The qPCR data showed a decrease in *Dhcr7*, *Lss*, and *Fdft1* gene expression, and the Western blot analysis showed decreased LSS and FDFT1 protein expression in lipid-accumulated HepG2 cells treated with ACT, revealing that ACT might restrain cholesterol synthesis by reducing the expression of these genes. ACAT is a key enzyme in the fat digestion and absorption pathway that resides in the endoplasmic reticulum of eukaryotic cells, where it catalyzes cholesterol synthesis and maintains the metabolic balance of intracellular cholesterol. Two isozymes of ACAT have been identified: ACAT1 and ACAT2. ACAT1 is universally expressed in humans, whereas ACAT2 is expressed in only the liver and small intestine and is involved in dietary cholesterol absorption and lipoprotein assembly. Overexpression of ACAT2 catalyzes the formation of fatty acid-cholesterol esters to produce foam cells that accumulate in vessel walls, leading to atherosclerosis and plaque formation (Pramfalk et al., 2007; Yin et al., 2014). Compared with the control group, the ACT group showed notably decreased expression of *Acat2*, revealing that ACT might improve lipid metabolism and synthesis by decreasing *Acat2* expression. HMGCR is the rate-limiting enzyme in cholesterol synthesis. In this experiment, HMGCR expression was significantly decreased in HepG2 cells treated with ACT compared with those in the model group, indicating that ACT likely regulates HMGCR levels to block endogenous liver cholesterol synthesis and exert its hypolipidemic effects (Peng et al., 2017). The HDL receptors SCARB1 and SCARB2 are key enzymes that mediate reverse cholesterol transport and promote cellular cholesterol efflux from peripheral cells and selective cholesterol uptake by the liver. Certain agonists increase the expression of these two genes to promote reverse cholesterol transport. Thus, SCARB1 and SCARB2 might be potential targets for the treatment of hyperlipidemia (Trigatti, 2017; Gillard et al., 2017). ACT might increase the expression of SCARB1 and SCARB2 to reduce cellular cholesterol accumulation. CPT1 is the rate limiting enzyme in fatty acid oxidation, and the CPT1A subtype is primarily expressed in the liver. CPT1A catalyzes the synthesis of fatty acyl carnitine from long-chain acyl-CoA and carnitine during fatty acid oxidation (Lee et al., 2011; Fontaine et al., 2012). SREBF1 plays an indispensable role in the synthesis and metabolism of fatty acids and cholesterol. SREBF1 overexpression activates multiple downstream enzymes, resulting in increased fatty acid synthesis. ACT might improve lipid metabolism and synthesis by influencing the expression of multiple genes, including *Scarb1*, *Scarb2*, *Srebf1*, *Dhcr7*, *Acat2*, *Hmgcr*, *Fdft1*, and *Lss*, involved in several pathways, such as glycolysis, antibiotic biosynthesis, AMPK signaling, and fatty acid degradation.

This study provides theoretical support for the relationships between a series of phenylethanoid glycosides represented by ACT and lipid metabolism and synthesis. The findings explain, to a certain extent, the mechanism by which ACT from *L. robustum* regulates lipid metabolism and synthesis, thus providing new prospects for the design and development of novel lipid-lowering drugs. However, the relationship between phenylethanoid glycosides and hypolipidemia is not simple. The relationships at the *in vivo* and *in vitro* levels must be identified, and to implement a more comprehensive study, the use of transcriptomic and proteomic analyses would reveal the overall relationship that varies from genes and mRNA to protein and regulatory factors.

## Author Contributions

LS, LX, BJ, and PX designed the experiments, supervised the study, and participated in the entire project. LS and FYu performed the experiments and contributed to data analysis, figure preparation, and the writing of the manuscript. FYi provided support for the *in silico* screening analysis. LL assisted with data analysis. All the authors read and approved the final manuscript.

## Funding

This work was supported by the National Natural Science Foundation of China (81573576) and the CAMS Innovation Fund for Medical Sciences (CIFMS; 2016-I2M-2-003).

## Conflict of Interest Statement

The authors declare that the research was conducted in the absence of any commercial or financial relationships that could be construed as a potential conflict of interest.
