# Supplementary material for: Acteoside From Ligustrum robustum (Roxb.) Blume Ameliorates Lipid Metabolism and Synthesis in a HepG2 Cell Model of Lipid Accumulation
Source: Front Pharmacol. 2019 May 24;10:602. doi: 10.3389/fphar.2019.00602 (PMC6543445; doi:10.3389/fphar.2019.00602)
Supplement: Supplementary file 1 [file Table_1.docx]

Supplementary file S1

**The structure of 13 phenylpropanoid glycosides from Ligustrum robustum (Roxb.) Blume**

1 ligurobustoside P, 2 ligurobustoside Q, 3 angoroside A, 4 Isoacteoside, 5 ligupurpuroside A, 6 acteoside, 7 ligupurpuroside B, 8 osmanthuside B, 9 osmanthuside B6, 10 ligupurpuroside C, 11 ligurobustoside M, 12 ligurobustoside N, 13 cis-ligupurpuroside B
