# Supplementary material for: Acteoside From Ligustrum robustum (Roxb.) Blume Ameliorates Lipid Metabolism and Synthesis in a HepG2 Cell Model of Lipid Accumulation
Source: Front Pharmacol. 2019 May 24;10:602. doi: 10.3389/fphar.2019.00602 (PMC6543445; doi:10.3389/fphar.2019.00602)
Supplement: Supplementary file 2 [file Table_2.docx]

Supplementary file S2

| Compound Name | Uniprot_ID | Gene Name | Fit Value | Shape Similarity |
| --- | --- | --- | --- | --- |
| 4 | P62942 | FKB1A | 0.565603 | 0.502604 |
| 8 | P62942 | FKB1A | 0.473308 | 0.503259 |
| 9 | P62942 | FKB1A | 0.439593 | 0.516556 |
| 6 | P62942 | FKB1A | 0.323929 | 0.508387 |
| 10 | P62942 | FKB1A | 0.240922 | 0.531496 |
| 11 | P00918 | CAH2 | 0.614658 | 0.525268 |
| 8 | P62942 | FKB1A | 0.632555 | 0.503448 |
| 10 | P62942 | FKB1A | 0.387101 | 0.501722 |
| 10 | P00734 | THRB | 0.148994 | 0.51004 |
| 11 | P48449 | ERG7 | 0.330248 | 0.505898 |
| 11 | Q9NXA8 | SIRT5 | 0.207995 | 0.534459 |
| 6 | P12931 | SRC | 0.242333 | 0.516556 |
| 4 | P00519 | ABL1 | 0.617601 | 0.520924 |
| 9 | P00519 | ABL1 | 0.129025 | 0.517391 |
| 4 | P56817 | BACE1 | 0.380823 | 0.515645 |
| 10 | P08254 | MMP3 | 0.25132 | 0.512445 |
| 10 | P52732 | KIF11 | 0.681783 | 0.509589 |
| 4 | P52732 | KIF11 | 0.482639 | 0.532236 |
| 11 | P08581 | MET | 0.390142 | 0.556222 |
| 9 | P11142 | HSP7C | 0.32885 | 0.518758 |
| 8 | P08069 | IGF1R | 0.759037 | 0.535966 |
| 11 | P00742 | FA10 | 0.295358 | 0.530899 |
| 6 | P48736 | PK3CG | 0.413762 | 0.503268 |
| 4 | P48736 | PK3CG | 0.127337 | 0.509485 |

**Table: The analysis result of virtual screening**
