# Supplementary material for: Acteoside From Ligustrum robustum (Roxb.) Blume Ameliorates Lipid Metabolism and Synthesis in a HepG2 Cell Model of Lipid Accumulation
Source: Front Pharmacol. 2019 May 24;10:602. doi: 10.3389/fphar.2019.00602 (PMC6543445; doi:10.3389/fphar.2019.00602)
Supplement: Supplementary file 3 [file Table_3.docx]

Supplementary file S3

| Gene ID | Gene Name | Model vs Control | | | AKG vs Model | | |
| --- | --- | --- | --- | --- | --- | --- | --- |
|  |  | log2FoldChange | Pvalue | Trend | log2FoldChange | Pvalue | Trend |
| ENSG00000271392 | RP1-161P9.5 | -3.02 | 0.05 | ↓ | 2.12 | 0.04 | ↑ |
| ENSG00000162654 | GBP4 | -2.79 | 0.03 | ↓ | 1.33 | 0.04 | ↑ |
| ENSG00000240338 | RP11-331F4.4 | -2.72 | 0.02 | ↓ | 1.33 | 0.04 | ↑ |
| ENSG00000261527 | RP11-343C2.10 | -2.71 | 0.04 | ↓ | 2.43 | 0.04 | ↑ |
| ENSG00000129673 | AANAT | -2.62 | 0.03 | ↓ | 3.24 | 0.03 | ↑ |
| ENSG00000267560 | RP11-173A16.1 | -2.37 | 0.01 | ↓ | 2.59 | 0.01 | ↑ |
| ENSG00000239827 | SUGT1P3 | -2.33 | 0.05 | ↓ | 3.28 | 0.01 | ↑ |
| ENSG00000169203 | RP11-231C14.4 | -2.21 | 0.01 | ↓ | 1.96 | 0.04 | ↑ |
| ENSG00000249532 | RP11-148B6.1 | -1.91 | 0.00 | ↓ | 1.60 | 0.00 | ↑ |
| ENSG00000260583 | AP000223.42 | -1.71 | 0.02 | ↓ | 2.00 | 0.04 | ↑ |
| ENSG00000172482 | AGXT | -1.70 | 0.04 | ↓ | 1.70 | 0.05 | ↑ |
| ENSG00000261669 | CTD-2515A14.1 | -1.66 | 0.04 | ↓ | 1.77 | 0.02 | ↑ |
| ENSG00000157150 | TIMP4 | -1.58 | 0.00 | ↓ | 1.03 | 0.05 | ↑ |
| ENSG00000172264 | MACROD2 | -1.37 | 0.04 | ↓ | 1.08 | 0.00 | ↑ |
| ENSG00000173762 | CD7 | -1.13 | 0.00 | ↓ | 0.37 | 0.02 | ↑ |
| ENSG00000229951 | FLJ31356 | -1.09 | 0.04 | ↓ | 1.43 | 0.03 | ↑ |
| ENSG00000277728 | RP11-143K11.7 | -1.08 | 0.02 | ↓ | 1.18 | 0.01 | ↑ |
| ENSG00000099194 | SCD | -1.06 | 0.00 | ↓ | 0.18 | 0.01 | ↑ |
| ENSG00000128917 | DLL4 | -1.00 | 0.01 | ↓ | 1.18 | 0.03 | ↑ |
| ENSG00000170801 | HTRA3 | -0.93 | 0.04 | ↓ | 0.51 | 0.04 | ↑ |
| ENSG00000274898 | AC001226.7 | -0.91 | 0.00 | ↓ | 1.01 | 0.00 | ↑ |
| ENSG00000158301 | GPRASP2 | -0.90 | 0.03 | ↓ | 0.83 | 0.03 | ↑ |
| ENSG00000151692 | RNF144A | -0.85 | 0.01 | ↓ | 0.73 | 0.01 | ↑ |
| ENSG00000260992 | DOCK9-AS2 | -0.83 | 0.01 | ↓ | 0.69 | 0.01 | ↑ |
| ENSG00000142149 | HUNK | -0.80 | 0.00 | ↓ | 0.99 | 0.00 | ↑ |
| ENSG00000127928 | GNGT1 | -0.79 | 0.04 | ↓ | 0.89 | 0.03 | ↑ |
| ENSG00000101198 | NKAIN4 | -0.79 | 0.04 | ↓ | 1.33 | 0.01 | ↑ |
| ENSG00000127561 | SYNGR3 | -0.78 | 0.00 | ↓ | 0.60 | 0.00 | ↑ |
| ENSG00000251532 | CTD-2245E15.3 | -0.75 | 0.01 | ↓ | 0.52 | 0.04 | ↑ |
| ENSG00000176894 | PXMP2 | -0.72 | 0.01 | ↓ | 0.52 | 0.03 | ↑ |
| ENSG00000234665 | RP11-262H14.3 | -0.71 | 0.05 | ↓ | 0.71 | 0.01 | ↑ |
| ENSG00000070915 | SLC12A3 | -0.70 | 0.00 | ↓ | 0.29 | 0.04 | ↑ |
| ENSG00000162526 | TSSK3 | -0.68 | 0.03 | ↓ | 0.60 | 0.04 | ↑ |
| ENSG00000087510 | TFAP2C | -0.66 | 0.02 | ↓ | 0.47 | 0.03 | ↑ |
| ENSG00000249395 | CASC9 | -0.66 | 0.04 | ↓ | 0.68 | 0.03 | ↑ |
| ENSG00000157601 | MX1 | -0.65 | 0.02 | ↓ | 0.33 | 0.03 | ↑ |
| ENSG00000277758 | ABC7-42404400C24.1 | -0.63 | 0.02 | ↓ | 0.48 | 0.02 | ↑ |
| ENSG00000089820 | ARHGAP4 | -0.62 | 0.02 | ↓ | 0.51 | 0.01 | ↑ |
| ENSG00000162645 | GBP2 | -0.61 | 0.04 | ↓ | 0.15 | 0.04 | ↑ |
| ENSG00000230555 | RP11-517P14.2 | -0.60 | 0.04 | ↓ | 0.44 | 0.00 | ↑ |
| ENSG00000106070 | GRB10 | -0.58 | 0.01 | ↓ | 0.25 | 0.03 | ↑ |
| ENSG00000138166 | DUSP5 | -0.57 | 0.01 | ↓ | 0.15 | 0.04 | ↑ |
| ENSG00000070388 | FGF22 | -0.57 | 0.01 | ↓ | 0.89 | 0.03 | ↑ |
| ENSG00000237753 | AC079922.3 | -0.55 | 0.04 | ↓ | 0.59 | 0.04 | ↑ |
| ENSG00000131471 | AOC3 | -0.55 | 0.03 | ↓ | 0.76 | 0.01 | ↑ |
| ENSG00000169871 | TRIM56 | -0.54 | 0.00 | ↓ | 0.12 | 0.04 | ↑ |
| ENSG00000170340 | B3GNT2 | -0.53 | 0.04 | ↓ | 0.14 | 0.01 | ↑ |
| ENSG00000157514 | TSC22D3 | -0.53 | 0.00 | ↓ | 0.24 | 0.00 | ↑ |
| ENSG00000113739 | STC2 | -0.51 | 0.01 | ↓ | 0.50 | 0.01 | ↑ |
| ENSG00000152137 | HSPB8 | -0.49 | 0.01 | ↓ | 0.22 | 0.03 | ↑ |
| ENSG00000273472 | RP11-102N12.3 | -0.48 | 0.01 | ↓ | 0.87 | 0.02 | ↑ |
| ENSG00000189223 | PAX8-AS1 | -0.46 | 0.02 | ↓ | 1.14 | 0.00 | ↑ |
| ENSG00000166575 | TMEM135 | -0.46 | 0.05 | ↓ | 0.40 | 0.03 | ↑ |
| ENSG00000100290 | BIK | -0.46 | 0.00 | ↓ | 1.15 | 0.04 | ↑ |
| ENSG00000235823 | OLMALINC | -0.45 | 0.00 | ↓ | 0.44 | 0.00 | ↑ |
| ENSG00000180628 | PCGF5 | -0.45 | 0.00 | ↓ | 0.03 | 0.02 | ↑ |
| ENSG00000268879 | IGFL1P1 | -0.43 | 0.01 | ↓ | 1.06 | 0.00 | ↑ |
| ENSG00000268621 | AC006262.5 | -0.43 | 0.03 | ↓ | 1.01 | 0.00 | ↑ |
| ENSG00000121064 | SCPEP1 | -0.43 | 0.02 | ↓ | 0.41 | 0.02 | ↑ |
| ENSG00000267221 | CTD-2132N18.2 | -0.43 | 0.04 | ↓ | 0.61 | 0.02 | ↑ |
| ENSG00000102081 | FMR1 | -0.42 | 0.00 | ↓ | 0.13 | 0.01 | ↑ |
| ENSG00000251661 | RP11-326C3.11 | -0.42 | 0.00 | ↓ | 0.77 | 0.02 | ↑ |
| ENSG00000136842 | TMOD1 | -0.42 | 0.01 | ↓ | 0.46 | 0.02 | ↑ |
| ENSG00000111859 | NEDD9 | -0.42 | 0.01 | ↓ | 0.38 | 0.01 | ↑ |
| ENSG00000261455 | LINC01003 | -0.41 | 0.02 | ↓ | 0.40 | 0.04 | ↑ |
| ENSG00000244041 | LINC01011 | -0.41 | 0.02 | ↓ | 0.62 | 0.01 | ↑ |
| ENSG00000117010 | ZNF684 | -0.40 | 0.04 | ↓ | 0.51 | 0.03 | ↑ |
| ENSG00000141664 | ZCCHC2 | -0.40 | 0.00 | ↓ | 0.17 | 0.02 | ↑ |
| ENSG00000147852 | VLDLR | -0.40 | 0.00 | ↓ | 0.73 | 0.01 | ↑ |
| ENSG00000111684 | LPCAT3 | -0.40 | 0.01 | ↓ | 0.51 | 0.01 | ↑ |
| ENSG00000115041 | KCNIP3 | -0.38 | 0.04 | ↓ | 0.30 | 0.03 | ↑ |
| ENSG00000167552 | TUBA1A | -0.37 | 0.02 | ↓ | 0.39 | 0.02 | ↑ |
| ENSG00000114268 | PFKFB4 | -0.36 | 0.03 | ↓ | 0.94 | 0.03 | ↑ |
| ENSG00000234745 | HLA-B | -0.36 | 0.00 | ↓ | 0.09 | 0.03 | ↑ |
| ENSG00000113369 | ARRDC3 | -0.36 | 0.00 | ↓ | 0.57 | 0.00 | ↑ |
| ENSG00000101400 | SNTA1 | -0.36 | 0.05 | ↓ | 0.55 | 0.02 | ↑ |
| ENSG00000239467 | AC007405.6 | -0.35 | 0.01 | ↓ | 0.52 | 0.02 | ↑ |
| ENSG00000137868 | STRA6 | -0.35 | 0.04 | ↓ | 0.34 | 0.01 | ↑ |
| ENSG00000183155 | RABIF | -0.35 | 0.00 | ↓ | 0.26 | 0.05 | ↑ |
| ENSG00000214174 | AMZ2P1 | -0.34 | 0.01 | ↓ | 0.51 | 0.00 | ↑ |
| ENSG00000100104 | SRRD | -0.33 | 0.01 | ↓ | 0.34 | 0.03 | ↑ |
| ENSG00000186198 | SLC51B | -0.33 | 0.01 | ↓ | 0.21 | 0.02 | ↑ |
| ENSG00000073331 | ALPK1 | -0.32 | 0.03 | ↓ | 0.38 | 0.05 | ↑ |
| ENSG00000131094 | C1QL1 | -0.32 | 0.01 | ↓ | 0.36 | 0.01 | ↑ |
| ENSG00000125378 |  | -0.32 | 0.00 | ↓ | 0.15 | 0.01 | ↑ |
| ENSG00000186787 | SPIN2B | -0.32 | 0.01 | ↓ | 0.29 | 0.00 | ↑ |
| ENSG00000198960 | ARMCX6 | -0.32 | 0.01 | ↓ | 0.30 | 0.03 | ↑ |
| ENSG00000280164 | CH507-254M2.3 | -0.32 | 0.04 | ↓ | 0.71 | 0.01 | ↑ |
| ENSG00000117266 | CDK18 | -0.31 | 0.03 | ↓ | 0.69 | 0.01 | ↑ |
| ENSG00000072310 | SREBF1 | -0.30 | 0.00 | ↓ | 0.61 | 0.00 | ↑ |
| ENSG00000148180 | GSN | -0.30 | 0.01 | ↓ | 0.36 | 0.00 | ↑ |
| ENSG00000206195 | DUXAP8 | -0.30 | 0.02 | ↓ | 0.29 | 0.02 | ↑ |
| ENSG00000212747 | FAM127C | -0.30 | 0.00 | ↓ | 0.13 | 0.03 | ↑ |
| ENSG00000260942 | CAPN10-AS1 | -0.30 | 0.02 | ↓ | 0.36 | 0.04 | ↑ |
| ENSG00000278828 | HIST1H3H | -0.30 | 0.05 | ↓ | 0.34 | 0.02 | ↑ |
| ENSG00000164603 | C7orf60 | -0.29 | 0.05 | ↓ | 0.98 | 0.01 | ↑ |
| ENSG00000173706 | HEG1 | -0.28 | 0.01 | ↓ | 0.13 | 0.04 | ↑ |
| ENSG00000178093 | TSSK6 | -0.28 | 0.04 | ↓ | 0.36 | 0.04 | ↑ |
| ENSG00000240682 | ISY1 | -0.28 | 0.02 | ↓ | 0.37 | 0.01 | ↑ |
| ENSG00000101986 | ABCD1 | -0.28 | 0.01 | ↓ | 0.19 | 0.04 | ↑ |
| ENSG00000162545 | CAMK2N1 | -0.27 | 0.01 | ↓ | 0.29 | 0.03 | ↑ |
| ENSG00000255874 | LINC00346 | -0.27 | 0.03 | ↓ | 0.29 | 0.01 | ↑ |
| ENSG00000138760 | SCARB2 | -0.27 | 0.00 | ↓ | 0.08 | 0.05 | ↑ |
| ENSG00000280187 | CTC-351M12.1 | -0.27 | 0.03 | ↓ | 0.32 | 0.00 | ↑ |
| ENSG00000175105 | ZNF654 | -0.27 | 0.05 | ↓ | 0.68 | 0.00 | ↑ |
| ENSG00000144476 | ACKR3 | -0.26 | 0.04 | ↓ | 0.64 | 0.02 | ↑ |
| ENSG00000156639 | ZFAND3 | -0.26 | 0.03 | ↓ | 0.13 | 0.00 | ↑ |
| ENSG00000066923 | STAG3 | -0.26 | 0.03 | ↓ | 0.39 | 0.02 | ↑ |
| ENSG00000173402 | DAG1 | -0.25 | 0.01 | ↓ | 0.09 | 0.02 | ↑ |
| ENSG00000148219 | ASTN2 | -0.24 | 0.03 | ↓ | 0.13 | 0.03 | ↑ |
| ENSG00000183049 | CAMK1D | -0.24 | 0.01 | ↓ | 0.10 | 0.03 | ↑ |
| ENSG00000142910 | TINAGL1 | -0.24 | 0.02 | ↓ | 0.35 | 0.03 | ↑ |
| ENSG00000078246 | TULP3 | -0.24 | 0.00 | ↓ | 0.11 | 0.02 | ↑ |
| ENSG00000159399 | HK2 | -0.23 | 0.03 | ↓ | 0.90 | 0.01 | ↑ |
| ENSG00000278784 | RP11-468E2.11 | -0.23 | 0.04 | ↓ | 0.79 | 0.00 | ↑ |
| ENSG00000196391 | ZNF774 | -0.22 | 0.00 | ↓ | 0.27 | 0.00 | ↑ |
| ENSG00000197747 | S100A10 | -0.22 | 0.02 | ↓ | 0.48 | 0.01 | ↑ |
| ENSG00000143207 | RFWD2 | -0.22 | 0.05 | ↓ | 0.22 | 0.02 | ↑ |
| ENSG00000112511 | PHF1 | -0.22 | 0.01 | ↓ | 0.09 | 0.01 | ↑ |
| ENSG00000273604 | C17orf96 | -0.22 | 0.00 | ↓ | 0.22 | 0.00 | ↑ |
| ENSG00000141376 | BCAS3 | -0.22 | 0.01 | ↓ | 0.22 | 0.00 | ↑ |
| ENSG00000108395 | TRIM37 | -0.21 | 0.00 | ↓ | 0.26 | 0.01 | ↑ |
| ENSG00000277877 | RP11-11N7.5 | -0.21 | 0.04 | ↓ | 0.31 | 0.05 | ↑ |
| ENSG00000132561 | MATN2 | -0.21 | 0.01 | ↓ | 0.14 | 0.01 | ↑ |
| ENSG00000137310 | TCF19 | -0.21 | 0.01 | ↓ | 0.14 | 0.03 | ↑ |
| ENSG00000275216 | RP11-54H7.4 | -0.20 | 0.00 | ↓ | 0.22 | 0.05 | ↑ |
| ENSG00000146278 | PNRC1 | -0.19 | 0.05 | ↓ | 0.32 | 0.02 | ↑ |
| ENSG00000135148 | TRAFD1 | -0.19 | 0.02 | ↓ | 0.18 | 0.03 | ↑ |
| ENSG00000107438 | PDLIM1 | -0.19 | 0.01 | ↓ | 0.11 | 0.03 | ↑ |
| ENSG00000171603 | CLSTN1 | -0.19 | 0.01 | ↓ | 0.14 | 0.01 | ↑ |
| ENSG00000172007 | RAB33B | -0.19 | 0.04 | ↓ | 0.22 | 0.01 | ↑ |
| ENSG00000136492 | BRIP1 | -0.19 | 0.01 | ↓ | 0.07 | 0.04 | ↑ |
| ENSG00000012660 | ELOVL5 | -0.19 | 0.03 | ↓ | 0.03 | 0.04 | ↑ |
| ENSG00000109107 | ALDOC | -0.19 | 0.05 | ↓ | 0.82 | 0.00 | ↑ |
| ENSG00000117899 | MESDC2 | -0.19 | 0.00 | ↓ | 0.13 | 0.02 | ↑ |
| ENSG00000154217 | PITPNC1 | -0.18 | 0.02 | ↓ | 0.15 | 0.00 | ↑ |
| ENSG00000179021 | C3orf38 | -0.18 | 0.00 | ↓ | 0.16 | 0.00 | ↑ |
| ENSG00000163872 | YEATS2 | -0.17 | 0.00 | ↓ | 0.48 | 0.00 | ↑ |
| ENSG00000126804 | ZBTB1 | -0.16 | 0.03 | ↓ | 0.34 | 0.01 | ↑ |
| ENSG00000134030 | CTIF | -0.16 | 0.00 | ↓ | 0.15 | 0.03 | ↑ |
| ENSG00000112715 | VEGFA | -0.16 | 0.01 | ↓ | 0.53 | 0.04 | ↑ |
| ENSG00000197555 | SIPA1L1 | -0.16 | 0.02 | ↓ | 0.10 | 0.03 | ↑ |
| ENSG00000073060 | SCARB1 | -0.16 | 0.05 | ↓ | 0.35 | 0.01 | ↑ |
| ENSG00000160183 | TMPRSS3 | -0.16 | 0.02 | ↓ | 0.19 | 0.03 | ↑ |
| ENSG00000261094 | RP11-355O1.11 | -0.16 | 0.03 | ↓ | 0.26 | 0.01 | ↑ |
| ENSG00000059728 | MXD1 | -0.16 | 0.00 | ↓ | 0.14 | 0.04 | ↑ |
| ENSG00000185875 | THNSL1 | -0.16 | 0.02 | ↓ | 0.52 | 0.01 | ↑ |
| ENSG00000214517 | PPME1 | -0.16 | 0.05 | ↓ | 0.23 | 0.02 | ↑ |
| ENSG00000168517 | HEXIM2 | -0.16 | 0.04 | ↓ | 0.54 | 0.05 | ↑ |
| ENSG00000145901 | TNIP1 | -0.15 | 0.03 | ↓ | 0.43 | 0.02 | ↑ |
| ENSG00000118197 | DDX59 | -0.15 | 0.03 | ↓ | 0.13 | 0.04 | ↑ |
| ENSG00000114978 | MOB1A | -0.15 | 0.04 | ↓ | 0.12 | 0.03 | ↑ |
| ENSG00000118495 | PLAGL1 | -0.15 | 0.02 | ↓ | 0.42 | 0.00 | ↑ |
| ENSG00000171960 | PPIH | -0.15 | 0.00 | ↓ | 0.11 | 0.01 | ↑ |
| ENSG00000169372 | CRADD | -0.14 | 0.04 | ↓ | 0.16 | 0.02 | ↑ |
| ENSG00000120451 | SNX19 | -0.14 | 0.03 | ↓ | 0.19 | 0.04 | ↑ |
| ENSG00000183283 | DAZAP2 | -0.14 | 0.00 | ↓ | 0.07 | 0.00 | ↑ |
| ENSG00000171848 | RRM2 | -0.14 | 0.01 | ↓ | 0.11 | 0.02 | ↑ |
| ENSG00000151883 | PARP8 | -0.13 | 0.00 | ↓ | 0.05 | 0.03 | ↑ |
| ENSG00000265972 | TXNIP | -0.13 | 0.04 | ↓ | 0.77 | 0.00 | ↑ |
| ENSG00000091651 | ORC6 | -0.13 | 0.02 | ↓ | 0.09 | 0.04 | ↑ |
| ENSG00000073969 | NSF | -0.13 | 0.00 | ↓ | 0.07 | 0.01 | ↑ |
| ENSG00000116641 | DOCK7 | -0.13 | 0.04 | ↓ | 0.16 | 0.01 | ↑ |
| ENSG00000180573 | HIST1H2AC | -0.12 | 0.05 | ↓ | 0.14 | 0.01 | ↑ |
| ENSG00000171208 | NETO2 | -0.12 | 0.04 | ↓ | 0.19 | 0.03 | ↑ |
| ENSG00000196730 | DAPK1 | -0.12 | 0.05 | ↓ | 0.13 | 0.04 | ↑ |
| ENSG00000197557 | TTC30A | -0.12 | 0.01 | ↓ | 0.40 | 0.01 | ↑ |
| ENSG00000255529 | POLR2M | -0.11 | 0.03 | ↓ | 0.05 | 0.00 | ↑ |
| ENSG00000171475 | WIPF2 | -0.10 | 0.01 | ↓ | 0.05 | 0.00 | ↑ |
| ENSG00000092853 | CLSPN | -0.10 | 0.04 | ↓ | 0.04 | 0.01 | ↑ |
| ENSG00000185022 | MAFF | -0.10 | 0.03 | ↓ | 0.22 | 0.01 | ↑ |
| ENSG00000117000 | RLF | -0.10 | 0.01 | ↓ | 0.38 | 0.04 | ↑ |
| ENSG00000171314 | PGAM1 | -0.09 | 0.04 | ↓ | 0.43 | 0.01 | ↑ |
| ENSG00000135766 | EGLN1 | -0.09 | 0.02 | ↓ | 0.78 | 0.01 | ↑ |
| ENSG00000187735 | TCEA1 | -0.09 | 0.01 | ↓ | 0.05 | 0.03 | ↑ |
| ENSG00000157800 | SLC37A3 | -0.08 | 0.04 | ↓ | 0.15 | 0.01 | ↑ |
| ENSG00000151693 | ASAP2 | -0.07 | 0.01 | ↓ | 0.28 | 0.01 | ↑ |
| ENSG00000176834 | VSIG10 | -0.07 | 0.01 | ↓ | 0.18 | 0.02 | ↑ |
| ENSG00000145979 | TBC1D7 | -0.07 | 0.05 | ↓ | 0.21 | 0.05 | ↑ |
| ENSG00000142192 | APP | -0.07 | 0.02 | ↓ | 0.15 | 0.00 | ↑ |
| ENSG00000126247 | CAPNS1 | -0.07 | 0.01 | ↓ | 0.08 | 0.02 | ↑ |
| ENSG00000164904 | ALDH7A1 | -0.06 | 0.02 | ↓ | 0.06 | 0.03 | ↑ |
| ENSG00000130707 | ASS1 | -0.05 | 0.02 | ↓ | 0.09 | 0.01 | ↑ |
| ENSG00000087111 | PIGS | -0.05 | 0.02 | ↓ | 0.09 | 0.02 | ↑ |
| ENSG00000169410 | PTPN9 | -0.04 | 0.01 | ↓ | 0.12 | 0.05 | ↑ |
| ENSG00000162736 | NCSTN | -0.04 | 0.01 | ↓ | 0.02 | 0.01 | ↑ |
| ENSG00000182973 | CNOT10 | 0.02 | 0.04 | ↑ | -0.13 | 0.02 | ↓ |
| ENSG00000113013 | HSPA9 | 0.02 | 0.04 | ↑ | -0.14 | 0.03 | ↓ |
| ENSG00000110321 | EIF4G2 | 0.02 | 0.02 | ↑ | -0.14 | 0.01 | ↓ |
| ENSG00000196419 | XRCC6 | 0.03 | 0.04 | ↑ | -0.12 | 0.01 | ↓ |
| ENSG00000159346 | ADIPOR1 | 0.03 | 0.01 | ↑ | -0.04 | 0.03 | ↓ |
| ENSG00000066322 | ELOVL1 | 0.03 | 0.04 | ↑ | -0.19 | 0.01 | ↓ |
| ENSG00000134283 | PPHLN1 | 0.04 | 0.04 | ↑ | -0.06 | 0.04 | ↓ |
| ENSG00000137804 | NUSAP1 | 0.04 | 0.04 | ↑ | -0.13 | 0.01 | ↓ |
| ENSG00000144366 | GULP1 | 0.04 | 0.01 | ↑ | -0.18 | 0.01 | ↓ |
| ENSG00000124207 | CSE1L | 0.04 | 0.00 | ↑ | -0.15 | 0.01 | ↓ |
| ENSG00000137203 | TFAP2A | 0.05 | 0.02 | ↑ | -0.22 | 0.00 | ↓ |
| ENSG00000182963 | GJC1 | 0.05 | 0.02 | ↑ | -0.06 | 0.02 | ↓ |
| ENSG00000131263 | RLIM | 0.05 | 0.04 | ↑ | -0.12 | 0.00 | ↓ |
| ENSG00000100297 | MCM5 | 0.05 | 0.05 | ↑ | -0.11 | 0.03 | ↓ |
| ENSG00000110318 | CEP126 | 0.06 | 0.01 | ↑ | -0.07 | 0.00 | ↓ |
| ENSG00000183624 | HMCES | 0.06 | 0.05 | ↑ | -0.13 | 0.02 | ↓ |
| ENSG00000136527 | TRA2B | 0.06 | 0.01 | ↑ | -0.16 | 0.04 | ↓ |
| ENSG00000152234 | ATP5A1 | 0.06 | 0.02 | ↑ | -0.08 | 0.00 | ↓ |
| ENSG00000048649 | RSF1 | 0.06 | 0.01 | ↑ | -0.08 | 0.01 | ↓ |
| ENSG00000156171 | DRAM2 | 0.06 | 0.03 | ↑ | -0.07 | 0.02 | ↓ |
| ENSG00000147955 | SIGMAR1 | 0.06 | 0.05 | ↑ | -0.12 | 0.04 | ↓ |
| ENSG00000006744 | ELAC2 | 0.06 | 0.02 | ↑ | -0.21 | 0.01 | ↓ |
| ENSG00000165732 | DDX21 | 0.07 | 0.05 | ↑ | -0.25 | 0.01 | ↓ |
| ENSG00000099812 | MISP | 0.07 | 0.03 | ↑ | -0.13 | 0.03 | ↓ |
| ENSG00000132842 | AP3B1 | 0.07 | 0.02 | ↑ | -0.18 | 0.00 | ↓ |
| ENSG00000154473 | BUB3 | 0.07 | 0.01 | ↑ | -0.06 | 0.02 | ↓ |
| ENSG00000004864 | SLC25A13 | 0.07 | 0.02 | ↑ | -0.08 | 0.01 | ↓ |
| ENSG00000136270 | TBRG4 | 0.07 | 0.03 | ↑ | -0.07 | 0.03 | ↓ |
| ENSG00000170144 | HNRNPA3 | 0.07 | 0.01 | ↑ | -0.08 | 0.01 | ↓ |
| ENSG00000113360 | DROSHA | 0.07 | 0.03 | ↑ | -0.03 | 0.01 | ↓ |
| ENSG00000136628 | EPRS | 0.07 | 0.03 | ↑ | -0.13 | 0.01 | ↓ |
| ENSG00000101161 | PRPF6 | 0.07 | 0.03 | ↑ | -0.06 | 0.04 | ↓ |
| ENSG00000148248 | SURF4 | 0.08 | 0.05 | ↑ | -0.12 | 0.01 | ↓ |
| ENSG00000121892 | PDS5A | 0.08 | 0.01 | ↑ | -0.16 | 0.04 | ↓ |
| ENSG00000105953 | OGDH | 0.08 | 0.01 | ↑ | -0.19 | 0.00 | ↓ |
| ENSG00000140564 | FURIN | 0.08 | 0.05 | ↑ | -0.12 | 0.02 | ↓ |
| ENSG00000120696 | KBTBD7 | 0.08 | 0.00 | ↑ | -0.32 | 0.00 | ↓ |
| ENSG00000265354 | TIMM23 | 0.08 | 0.02 | ↑ | -0.17 | 0.00 | ↓ |
| ENSG00000157778 | PSMG3 | 0.08 | 0.03 | ↑ | -0.14 | 0.03 | ↓ |
| ENSG00000144028 | SNRNP200 | 0.08 | 0.02 | ↑ | -0.03 | 0.00 | ↓ |
| ENSG00000065978 | YBX1 | 0.08 | 0.01 | ↑ | -0.10 | 0.00 | ↓ |
| ENSG00000120948 | TARDBP | 0.08 | 0.03 | ↑ | -0.10 | 0.04 | ↓ |
| ENSG00000142864 | SERBP1 | 0.08 | 0.00 | ↑ | -0.03 | 0.03 | ↓ |
| ENSG00000117523 | PRRC2C | 0.08 | 0.03 | ↑ | -0.11 | 0.00 | ↓ |
| ENSG00000170677 | SOCS6 | 0.08 | 0.01 | ↑ | -0.16 | 0.04 | ↓ |
| ENSG00000181163 | NPM1 | 0.08 | 0.01 | ↑ | -0.13 | 0.02 | ↓ |
| ENSG00000123505 | AMD1 | 0.08 | 0.00 | ↑ | -0.36 | 0.01 | ↓ |
| ENSG00000099958 | DERL3 | 0.08 | 0.02 | ↑ | -0.64 | 0.00 | ↓ |
| ENSG00000140403 | DNAJA4 | 0.09 | 0.04 | ↑ | -0.33 | 0.00 | ↓ |
| ENSG00000134910 | STT3A | 0.09 | 0.04 | ↑ | -0.04 | 0.03 | ↓ |
| ENSG00000162851 | TFB2M | 0.09 | 0.00 | ↑ | -0.23 | 0.00 | ↓ |
| ENSG00000075292 | ZNF638 | 0.09 | 0.03 | ↑ | -0.09 | 0.01 | ↓ |
| ENSG00000155506 | LARP1 | 0.09 | 0.04 | ↑ | -0.14 | 0.02 | ↓ |
| ENSG00000117650 | NEK2 | 0.09 | 0.00 | ↑ | -0.17 | 0.00 | ↓ |
| ENSG00000116584 | ARHGEF2 | 0.09 | 0.04 | ↑ | -0.19 | 0.01 | ↓ |
| ENSG00000179218 | CALR | 0.09 | 0.01 | ↑ | -0.04 | 0.02 | ↓ |
| ENSG00000184009 | ACTG1 | 0.10 | 0.01 | ↑ | -0.12 | 0.00 | ↓ |
| ENSG00000088899 | RP5-1187M17.10 | 0.10 | 0.00 | ↑ | -0.15 | 0.00 | ↓ |
| ENSG00000132600 | PRMT7 | 0.10 | 0.02 | ↑ | -0.10 | 0.02 | ↓ |
| ENSG00000033050 | ABCF2 | 0.10 | 0.04 | ↑ | -0.22 | 0.00 | ↓ |
| ENSG00000111642 | CHD4 | 0.10 | 0.00 | ↑ | -0.24 | 0.00 | ↓ |
| ENSG00000101413 | RPRD1B | 0.10 | 0.00 | ↑ | -0.06 | 0.02 | ↓ |
| ENSG00000132357 | CARD6 | 0.10 | 0.02 | ↑ | -0.16 | 0.00 | ↓ |
| ENSG00000108179 | PPIF | 0.10 | 0.01 | ↑ | -0.16 | 0.02 | ↓ |
| ENSG00000116560 | SFPQ | 0.10 | 0.01 | ↑ | -0.21 | 0.00 | ↓ |
| ENSG00000115677 | HDLBP | 0.10 | 0.00 | ↑ | -0.04 | 0.04 | ↓ |
| ENSG00000197451 | HNRNPAB | 0.10 | 0.04 | ↑ | -0.14 | 0.02 | ↓ |
| ENSG00000115355 | CCDC88A | 0.10 | 0.04 | ↑ | -0.21 | 0.02 | ↓ |
| ENSG00000065154 | OAT | 0.10 | 0.00 | ↑ | -0.13 | 0.03 | ↓ |
| ENSG00000129250 | KIF1C | 0.10 | 0.03 | ↑ | -0.07 | 0.00 | ↓ |
| ENSG00000114686 | MRPL3 | 0.10 | 0.03 | ↑ | -0.16 | 0.04 | ↓ |
| ENSG00000153187 | HNRNPU | 0.10 | 0.04 | ↑ | -0.15 | 0.01 | ↓ |
| ENSG00000117222 | RBBP5 | 0.11 | 0.04 | ↑ | -0.18 | 0.03 | ↓ |
| ENSG00000144747 | TMF1 | 0.11 | 0.02 | ↑ | -0.18 | 0.01 | ↓ |
| ENSG00000133316 | WDR74 | 0.11 | 0.04 | ↑ | -0.22 | 0.00 | ↓ |
| ENSG00000130939 | UBE4B | 0.11 | 0.04 | ↑ | -0.17 | 0.03 | ↓ |
| ENSG00000083642 | PDS5B | 0.11 | 0.03 | ↑ | -0.08 | 0.02 | ↓ |
| ENSG00000184216 | IRAK1 | 0.11 | 0.00 | ↑ | -0.08 | 0.04 | ↓ |
| ENSG00000134697 | GNL2 | 0.11 | 0.04 | ↑ | -0.09 | 0.05 | ↓ |
| ENSG00000023516 | AKAP11 | 0.11 | 0.01 | ↑ | -0.17 | 0.00 | ↓ |
| ENSG00000103994 | ZNF106 | 0.11 | 0.05 | ↑ | -0.12 | 0.04 | ↓ |
| ENSG00000138385 | SSB | 0.11 | 0.03 | ↑ | -0.18 | 0.01 | ↓ |
| ENSG00000047315 | POLR2B | 0.11 | 0.03 | ↑ | -0.11 | 0.02 | ↓ |
| ENSG00000120800 | UTP20 | 0.11 | 0.00 | ↑ | -0.28 | 0.00 | ↓ |
| ENSG00000152409 | JMY | 0.11 | 0.04 | ↑ | -0.10 | 0.01 | ↓ |
| ENSG00000136485 | DCAF7 | 0.11 | 0.02 | ↑ | -0.09 | 0.05 | ↓ |
| ENSG00000102317 | RBM3 | 0.11 | 0.02 | ↑ | -0.13 | 0.01 | ↓ |
| ENSG00000134690 | CDCA8 | 0.11 | 0.01 | ↑ | -0.12 | 0.01 | ↓ |
| ENSG00000085224 | ATRX | 0.11 | 0.00 | ↑ | -0.12 | 0.02 | ↓ |
| ENSG00000102225 | CDK16 | 0.12 | 0.02 | ↑ | -0.02 | 0.02 | ↓ |
| ENSG00000198818 | SFT2D1 | 0.12 | 0.00 | ↑ | -0.11 | 0.02 | ↓ |
| ENSG00000273611 | ZNHIT3 | 0.12 | 0.04 | ↑ | -0.10 | 0.04 | ↓ |
| ENSG00000136045 | PWP1 | 0.12 | 0.05 | ↑ | -0.21 | 0.00 | ↓ |
| ENSG00000101182 | PSMA7 | 0.12 | 0.04 | ↑ | -0.06 | 0.04 | ↓ |
| ENSG00000124299 | PEPD | 0.12 | 0.03 | ↑ | -0.08 | 0.04 | ↓ |
| ENSG00000171681 | ATF7IP | 0.12 | 0.01 | ↑ | -0.43 | 0.00 | ↓ |
| ENSG00000169895 | SYAP1 | 0.12 | 0.00 | ↑ | -0.08 | 0.05 | ↓ |
| ENSG00000083520 | DIS3 | 0.12 | 0.03 | ↑ | -0.14 | 0.01 | ↓ |
| ENSG00000167258 | CDK12 | 0.12 | 0.03 | ↑ | -0.16 | 0.01 | ↓ |
| ENSG00000135916 | ITM2C | 0.12 | 0.03 | ↑ | -0.08 | 0.03 | ↓ |
| ENSG00000165219 | GAPVD1 | 0.12 | 0.03 | ↑ | -0.10 | 0.00 | ↓ |
| ENSG00000135046 | ANXA1 | 0.12 | 0.03 | ↑ | -0.22 | 0.01 | ↓ |
| ENSG00000088325 | TPX2 | 0.12 | 0.01 | ↑ | -0.18 | 0.01 | ↓ |
| ENSG00000089053 | ANAPC5 | 0.12 | 0.01 | ↑ | -0.11 | 0.05 | ↓ |
| ENSG00000157456 | CCNB2 | 0.12 | 0.01 | ↑ | -0.17 | 0.02 | ↓ |
| ENSG00000091127 | PUS7 | 0.12 | 0.05 | ↑ | -0.10 | 0.02 | ↓ |
| ENSG00000116459 | ATP5F1 | 0.12 | 0.02 | ↑ | -0.12 | 0.03 | ↓ |
| ENSG00000033011 | ALG1 | 0.13 | 0.01 | ↑ | -0.29 | 0.00 | ↓ |
| ENSG00000100401 | RANGAP1 | 0.13 | 0.03 | ↑ | -0.18 | 0.02 | ↓ |
| ENSG00000125753 | VASP | 0.13 | 0.00 | ↑ | -0.09 | 0.03 | ↓ |
| ENSG00000068878 | PSME4 | 0.13 | 0.05 | ↑ | -0.19 | 0.03 | ↓ |
| ENSG00000196290 | NIF3L1 | 0.13 | 0.01 | ↑ | -0.08 | 0.04 | ↓ |
| ENSG00000165322 | ARHGAP12 | 0.13 | 0.04 | ↑ | -0.31 | 0.03 | ↓ |
| ENSG00000148843 | PDCD11 | 0.13 | 0.01 | ↑ | -0.08 | 0.02 | ↓ |
| ENSG00000085662 | AKR1B1 | 0.13 | 0.04 | ↑ | -0.25 | 0.01 | ↓ |
| ENSG00000056097 | ZFR | 0.13 | 0.02 | ↑ | -0.20 | 0.03 | ↓ |
| ENSG00000113812 | ACTR8 | 0.13 | 0.03 | ↑ | -0.25 | 0.02 | ↓ |
| ENSG00000138835 | RGS3 | 0.13 | 0.04 | ↑ | -0.38 | 0.01 | ↓ |
| ENSG00000103404 | USP31 | 0.13 | 0.00 | ↑ | -0.10 | 0.01 | ↓ |
| ENSG00000196535 | MYO18A | 0.13 | 0.03 | ↑ | -0.09 | 0.04 | ↓ |
| ENSG00000101391 | CDK5RAP1 | 0.13 | 0.00 | ↑ | -0.14 | 0.01 | ↓ |
| ENSG00000011009 | LYPLA2 | 0.13 | 0.03 | ↑ | -0.16 | 0.03 | ↓ |
| ENSG00000107581 | EIF3A | 0.13 | 0.01 | ↑ | -0.21 | 0.01 | ↓ |
| ENSG00000136231 | IGF2BP3 | 0.13 | 0.01 | ↑ | -0.11 | 0.01 | ↓ |
| ENSG00000213965 | NUDT19 | 0.14 | 0.03 | ↑ | -0.09 | 0.03 | ↓ |
| ENSG00000137955 | RABGGTB | 0.14 | 0.01 | ↑ | -0.15 | 0.01 | ↓ |
| ENSG00000072864 | NDE1 | 0.14 | 0.02 | ↑ | -0.28 | 0.01 | ↓ |
| ENSG00000162694 | EXTL2 | 0.14 | 0.05 | ↑ | -0.24 | 0.01 | ↓ |
| ENSG00000087074 | PPP1R15A | 0.14 | 0.01 | ↑ | -0.17 | 0.01 | ↓ |
| ENSG00000156256 | USP16 | 0.14 | 0.04 | ↑ | -0.20 | 0.02 | ↓ |
| ENSG00000198162 | MAN1A2 | 0.14 | 0.02 | ↑ | -0.04 | 0.04 | ↓ |
| ENSG00000136891 | TEX10 | 0.14 | 0.00 | ↑ | -0.33 | 0.03 | ↓ |
| ENSG00000117597 | DIEXF | 0.14 | 0.01 | ↑ | -0.10 | 0.05 | ↓ |
| ENSG00000099783 | HNRNPM | 0.14 | 0.01 | ↑ | -0.08 | 0.04 | ↓ |
| ENSG00000123146 | ADGRE5 | 0.14 | 0.01 | ↑ | -0.21 | 0.01 | ↓ |
| ENSG00000179889 | PDXDC1 | 0.14 | 0.03 | ↑ | -0.15 | 0.02 | ↓ |
| ENSG00000116199 | FAM20B | 0.14 | 0.03 | ↑ | -0.13 | 0.03 | ↓ |
| ENSG00000161800 | RACGAP1 | 0.14 | 0.04 | ↑ | -0.11 | 0.05 | ↓ |
| ENSG00000124588 | NQO2 | 0.14 | 0.02 | ↑ | -0.19 | 0.03 | ↓ |
| ENSG00000119403 | PHF19 | 0.14 | 0.02 | ↑ | -0.13 | 0.02 | ↓ |
| ENSG00000101447 | FAM83D | 0.14 | 0.00 | ↑ | -0.27 | 0.04 | ↓ |
| ENSG00000164338 | UTP15 | 0.14 | 0.01 | ↑ | -0.19 | 0.03 | ↓ |
| ENSG00000128595 | CALU | 0.14 | 0.01 | ↑ | -0.09 | 0.05 | ↓ |
| ENSG00000131323 | TRAF3 | 0.14 | 0.01 | ↑ | -0.08 | 0.01 | ↓ |
| ENSG00000123064 | DDX54 | 0.14 | 0.03 | ↑ | -0.05 | 0.02 | ↓ |
| ENSG00000154945 | ANKRD40 | 0.14 | 0.04 | ↑ | -0.25 | 0.03 | ↓ |
| ENSG00000205060 | SLC35B4 | 0.15 | 0.01 | ↑ | -0.27 | 0.01 | ↓ |
| ENSG00000130559 | CAMSAP1 | 0.15 | 0.01 | ↑ | -0.09 | 0.01 | ↓ |
| ENSG00000090905 | TNRC6A | 0.15 | 0.02 | ↑ | -0.09 | 0.00 | ↓ |
| ENSG00000138182 | KIF20B | 0.15 | 0.01 | ↑ | -0.19 | 0.04 | ↓ |
| ENSG00000126391 | FRMD8 | 0.15 | 0.00 | ↑ | -0.03 | 0.02 | ↓ |
| ENSG00000060339 | CCAR1 | 0.15 | 0.02 | ↑ | -0.18 | 0.02 | ↓ |
| ENSG00000182831 | C16orf72 | 0.15 | 0.03 | ↑ | -0.26 | 0.02 | ↓ |
| ENSG00000133816 | MICAL2 | 0.15 | 0.00 | ↑ | -0.35 | 0.03 | ↓ |
| ENSG00000181789 | COPG1 | 0.15 | 0.03 | ↑ | -0.23 | 0.02 | ↓ |
| ENSG00000141569 | TRIM65 | 0.15 | 0.04 | ↑ | -0.10 | 0.01 | ↓ |
| ENSG00000021776 | AQR | 0.15 | 0.03 | ↑ | -0.14 | 0.03 | ↓ |
| ENSG00000187951 | ARHGAP11B | 0.15 | 0.00 | ↑ | -0.15 | 0.03 | ↓ |
| ENSG00000123975 | CKS2 | 0.15 | 0.05 | ↑ | -0.21 | 0.03 | ↓ |
| ENSG00000142507 | PSMB6 | 0.15 | 0.02 | ↑ | -0.09 | 0.05 | ↓ |
| ENSG00000150764 | DIXDC1 | 0.15 | 0.02 | ↑ | -0.32 | 0.02 | ↓ |
| ENSG00000132300 | PTCD3 | 0.15 | 0.01 | ↑ | -0.16 | 0.01 | ↓ |
| ENSG00000181019 | NQO1 | 0.15 | 0.01 | ↑ | -0.25 | 0.00 | ↓ |
| ENSG00000198826 | ARHGAP11A | 0.16 | 0.00 | ↑ | -0.23 | 0.00 | ↓ |
| ENSG00000135778 | NTPCR | 0.16 | 0.03 | ↑ | -0.21 | 0.04 | ↓ |
| ENSG00000136271 | DDX56 | 0.16 | 0.04 | ↑ | -0.17 | 0.01 | ↓ |
| ENSG00000154734 | ADAMTS1 | 0.16 | 0.03 | ↑ | -0.41 | 0.02 | ↓ |
| ENSG00000158711 | ELK4 | 0.16 | 0.04 | ↑ | -0.17 | 0.05 | ↓ |
| ENSG00000108587 | GOSR1 | 0.16 | 0.00 | ↑ | -0.15 | 0.05 | ↓ |
| ENSG00000075711 | DLG1 | 0.16 | 0.00 | ↑ | -0.10 | 0.02 | ↓ |
| ENSG00000164062 | APEH | 0.16 | 0.00 | ↑ | -0.23 | 0.00 | ↓ |
| ENSG00000089685 | BIRC5 | 0.16 | 0.00 | ↑ | -0.08 | 0.01 | ↓ |
| ENSG00000132153 | DHX30 | 0.16 | 0.02 | ↑ | -0.10 | 0.04 | ↓ |
| ENSG00000178695 | KCTD12 | 0.16 | 0.04 | ↑ | -0.18 | 0.02 | ↓ |
| ENSG00000114503 | NCBP2 | 0.16 | 0.00 | ↑ | -0.12 | 0.00 | ↓ |
| ENSG00000023839 | ABCC2 | 0.16 | 0.04 | ↑ | -0.27 | 0.02 | ↓ |
| ENSG00000148344 | PTGES | 0.17 | 0.01 | ↑ | -0.26 | 0.02 | ↓ |
| ENSG00000076382 | SPAG5 | 0.17 | 0.02 | ↑ | -0.17 | 0.02 | ↓ |
| ENSG00000204842 | ATXN2 | 0.17 | 0.03 | ↑ | -0.15 | 0.01 | ↓ |
| ENSG00000176390 | CRLF3 | 0.17 | 0.02 | ↑ | -0.10 | 0.05 | ↓ |
| ENSG00000169629 | RGPD8 | 0.17 | 0.03 | ↑ | -0.42 | 0.02 | ↓ |
| ENSG00000164403 | SHROOM1 | 0.17 | 0.03 | ↑ | -0.14 | 0.02 | ↓ |
| ENSG00000100304 | TTLL12 | 0.17 | 0.00 | ↑ | -0.09 | 0.03 | ↓ |
| ENSG00000141934 | PLPP2 | 0.17 | 0.00 | ↑ | -0.18 | 0.00 | ↓ |
| ENSG00000175467 | SART1 | 0.17 | 0.02 | ↑ | -0.22 | 0.03 | ↓ |
| ENSG00000112640 | PPP2R5D | 0.17 | 0.01 | ↑ | -0.16 | 0.01 | ↓ |
| ENSG00000085733 | CTTN | 0.18 | 0.03 | ↑ | -0.08 | 0.03 | ↓ |
| ENSG00000142002 | DPP9 | 0.18 | 0.01 | ↑ | -0.10 | 0.04 | ↓ |
| ENSG00000007038 | PRSS21 | 0.18 | 0.00 | ↑ | -0.11 | 0.01 | ↓ |
| ENSG00000148300 | REXO4 | 0.18 | 0.00 | ↑ | -0.09 | 0.00 | ↓ |
| ENSG00000141068 | KSR1 | 0.18 | 0.01 | ↑ | -0.27 | 0.02 | ↓ |
| ENSG00000102078 | SLC25A14 | 0.18 | 0.01 | ↑ | -0.17 | 0.02 | ↓ |
| ENSG00000100263 | RHBDD3 | 0.18 | 0.05 | ↑ | -0.20 | 0.01 | ↓ |
| ENSG00000120662 | MTRF1 | 0.18 | 0.03 | ↑ | -0.14 | 0.05 | ↓ |
| ENSG00000143815 | LBR | 0.18 | 0.02 | ↑ | -0.23 | 0.03 | ↓ |
| ENSG00000118777 | ABCG2 | 0.18 | 0.01 | ↑ | -0.24 | 0.01 | ↓ |
| ENSG00000151914 | DST | 0.18 | 0.01 | ↑ | -0.49 | 0.01 | ↓ |
| ENSG00000134057 | CCNB1 | 0.19 | 0.02 | ↑ | -0.30 | 0.00 | ↓ |
| ENSG00000137216 | TMEM63B | 0.19 | 0.00 | ↑ | -0.18 | 0.00 | ↓ |
| ENSG00000163166 | IWS1 | 0.19 | 0.01 | ↑ | -0.17 | 0.04 | ↓ |
| ENSG00000179295 | PTPN11 | 0.19 | 0.02 | ↑ | -0.18 | 0.03 | ↓ |
| ENSG00000146834 | MEPCE | 0.19 | 0.02 | ↑ | -0.17 | 0.01 | ↓ |
| ENSG00000198901 | PRC1 | 0.19 | 0.01 | ↑ | -0.10 | 0.04 | ↓ |
| ENSG00000165689 | SDCCAG3 | 0.19 | 0.01 | ↑ | -0.09 | 0.02 | ↓ |
| ENSG00000074071 | MRPS34 | 0.19 | 0.02 | ↑ | -0.14 | 0.01 | ↓ |
| ENSG00000167880 | EVPL | 0.19 | 0.01 | ↑ | -0.23 | 0.04 | ↓ |
| ENSG00000167700 | MFSD3 | 0.19 | 0.03 | ↑ | -0.15 | 0.02 | ↓ |
| ENSG00000125746 | EML2 | 0.19 | 0.01 | ↑ | -0.35 | 0.01 | ↓ |
| ENSG00000087586 | AURKA | 0.20 | 0.02 | ↑ | -0.40 | 0.01 | ↓ |
| ENSG00000162191 | UBXN1 | 0.20 | 0.02 | ↑ | -0.26 | 0.02 | ↓ |
| ENSG00000113758 | DBN1 | 0.20 | 0.01 | ↑ | -0.16 | 0.01 | ↓ |
| ENSG00000148187 | MRRF | 0.20 | 0.02 | ↑ | -0.13 | 0.03 | ↓ |
| ENSG00000149091 | DGKZ | 0.20 | 0.02 | ↑ | -0.22 | 0.01 | ↓ |
| ENSG00000136718 | IMP4 | 0.20 | 0.04 | ↑ | -0.18 | 0.03 | ↓ |
| ENSG00000006695 | COX10 | 0.20 | 0.01 | ↑ | -0.12 | 0.02 | ↓ |
| ENSG00000179348 | GATA2 | 0.20 | 0.03 | ↑ | -0.23 | 0.03 | ↓ |
| ENSG00000167553 | TUBA1C | 0.20 | 0.03 | ↑ | -0.29 | 0.02 | ↓ |
| ENSG00000156253 | RWDD2B | 0.21 | 0.01 | ↑ | -0.15 | 0.04 | ↓ |
| ENSG00000166845 | C18orf54 | 0.21 | 0.02 | ↑ | -0.20 | 0.04 | ↓ |
| ENSG00000169019 | COMMD8 | 0.21 | 0.05 | ↑ | -0.27 | 0.04 | ↓ |
| ENSG00000166263 | STXBP4 | 0.21 | 0.01 | ↑ | -0.17 | 0.01 | ↓ |
| ENSG00000117399 | CDC20 | 0.21 | 0.01 | ↑ | -0.34 | 0.01 | ↓ |
| ENSG00000186376 | ZNF75D | 0.21 | 0.05 | ↑ | -0.23 | 0.05 | ↓ |
| ENSG00000104884 | ERCC2 | 0.21 | 0.02 | ↑ | -0.17 | 0.04 | ↓ |
| ENSG00000071626 | DAZAP1 | 0.22 | 0.03 | ↑ | -0.10 | 0.04 | ↓ |
| ENSG00000105197 | TIMM50 | 0.22 | 0.01 | ↑ | -0.20 | 0.01 | ↓ |
| ENSG00000146223 | RPL7L1 | 0.22 | 0.00 | ↑ | -0.10 | 0.01 | ↓ |
| ENSG00000110046 | ATG2A | 0.22 | 0.00 | ↑ | -0.38 | 0.00 | ↓ |
| ENSG00000183020 | AP2A2 | 0.22 | 0.01 | ↑ | -0.07 | 0.04 | ↓ |
| ENSG00000112984 | KIF20A | 0.22 | 0.00 | ↑ | -0.15 | 0.01 | ↓ |
| ENSG00000123600 | METTL8 | 0.22 | 0.02 | ↑ | -0.18 | 0.02 | ↓ |
| ENSG00000177156 | TALDO1 | 0.22 | 0.01 | ↑ | -0.13 | 0.01 | ↓ |
| ENSG00000183605 | SFXN4 | 0.22 | 0.00 | ↑ | -0.09 | 0.01 | ↓ |
| ENSG00000181027 | FKRP | 0.22 | 0.01 | ↑ | -0.12 | 0.04 | ↓ |
| ENSG00000153714 | LURAP1L | 0.22 | 0.00 | ↑ | -0.39 | 0.03 | ↓ |
| ENSG00000008283 | CYB561 | 0.22 | 0.01 | ↑ | -0.42 | 0.00 | ↓ |
| ENSG00000169718 | DUS1L | 0.22 | 0.01 | ↑ | -0.12 | 0.02 | ↓ |
| ENSG00000204946 | ZNF783 | 0.23 | 0.05 | ↑ | -0.25 | 0.00 | ↓ |
| ENSG00000187630 | DHRS4L2 | 0.23 | 0.04 | ↑ | -0.18 | 0.05 | ↓ |
| ENSG00000122218 | COPA | 0.23 | 0.00 | ↑ | -0.14 | 0.01 | ↓ |
| ENSG00000185989 | RASA3 | 0.23 | 0.00 | ↑ | -0.38 | 0.02 | ↓ |
| ENSG00000115163 | CENPA | 0.23 | 0.04 | ↑ | -0.26 | 0.02 | ↓ |
| ENSG00000174851 | YIF1A | 0.23 | 0.02 | ↑ | -0.23 | 0.02 | ↓ |
| ENSG00000105676 | ARMC6 | 0.23 | 0.05 | ↑ | -0.24 | 0.05 | ↓ |
| ENSG00000117139 | KDM5B | 0.23 | 0.00 | ↑ | -0.05 | 0.04 | ↓ |
| ENSG00000116016 | EPAS1 | 0.23 | 0.00 | ↑ | -0.05 | 0.01 | ↓ |
| ENSG00000160049 | DFFA | 0.23 | 0.01 | ↑ | -0.12 | 0.04 | ↓ |
| ENSG00000131747 | TOP2A | 0.23 | 0.01 | ↑ | -0.09 | 0.01 | ↓ |
| ENSG00000165525 | NEMF | 0.23 | 0.00 | ↑ | -0.18 | 0.01 | ↓ |
| ENSG00000162063 | CCNF | 0.23 | 0.04 | ↑ | -0.11 | 0.05 | ↓ |
| ENSG00000167778 | SPRYD3 | 0.24 | 0.00 | ↑ | -0.36 | 0.00 | ↓ |
| ENSG00000105538 | RASIP1 | 0.24 | 0.01 | ↑ | -0.21 | 0.03 | ↓ |
| ENSG00000247092 | SNHG10 | 0.24 | 0.04 | ↑ | -0.44 | 0.03 | ↓ |
| ENSG00000122778 | KIAA1549 | 0.24 | 0.01 | ↑ | -0.17 | 0.00 | ↓ |
| ENSG00000197712 | FAM114A1 | 0.24 | 0.02 | ↑ | -0.12 | 0.03 | ↓ |
| ENSG00000101361 | NOP56 | 0.24 | 0.02 | ↑ | -0.20 | 0.03 | ↓ |
| ENSG00000117724 | CENPF | 0.24 | 0.01 | ↑ | -0.23 | 0.01 | ↓ |
| ENSG00000105127 | AKAP8 | 0.24 | 0.03 | ↑ | -0.19 | 0.02 | ↓ |
| ENSG00000005156 | LIG3 | 0.24 | 0.01 | ↑ | -0.20 | 0.01 | ↓ |
| ENSG00000132382 | MYBBP1A | 0.24 | 0.00 | ↑ | -0.17 | 0.00 | ↓ |
| ENSG00000174989 | FBXW8 | 0.25 | 0.03 | ↑ | -0.25 | 0.04 | ↓ |
| ENSG00000136877 | FPGS | 0.25 | 0.01 | ↑ | -0.21 | 0.02 | ↓ |
| ENSG00000183092 | BEGAIN | 0.25 | 0.01 | ↑ | -0.25 | 0.01 | ↓ |
| ENSG00000010292 | NCAPD2 | 0.25 | 0.00 | ↑ | -0.06 | 0.03 | ↓ |
| ENSG00000214160 | ALG3 | 0.25 | 0.00 | ↑ | -0.13 | 0.03 | ↓ |
| ENSG00000136811 | ODF2 | 0.25 | 0.00 | ↑ | -0.10 | 0.05 | ↓ |
| ENSG00000072571 | HMMR | 0.25 | 0.04 | ↑ | -0.20 | 0.01 | ↓ |
| ENSG00000162062 | C16orf59 | 0.26 | 0.00 | ↑ | -0.13 | 0.02 | ↓ |
| ENSG00000115758 | ODC1 | 0.26 | 0.00 | ↑ | -0.37 | 0.01 | ↓ |
| ENSG00000117308 | GALE | 0.26 | 0.00 | ↑ | -0.29 | 0.03 | ↓ |
| ENSG00000196236 | XPNPEP3 | 0.26 | 0.01 | ↑ | -0.32 | 0.03 | ↓ |
| ENSG00000090674 | MCOLN1 | 0.26 | 0.04 | ↑ | -0.26 | 0.00 | ↓ |
| ENSG00000161011 | SQSTM1 | 0.26 | 0.01 | ↑ | -0.12 | 0.03 | ↓ |
| ENSG00000169282 | KCNAB1 | 0.26 | 0.01 | ↑ | -0.25 | 0.03 | ↓ |
| ENSG00000138778 | CENPE | 0.26 | 0.03 | ↑ | -0.44 | 0.01 | ↓ |
| ENSG00000161647 | MPP3 | 0.26 | 0.01 | ↑ | -0.15 | 0.04 | ↓ |
| ENSG00000175920 | DOK7 | 0.27 | 0.00 | ↑ | -0.27 | 0.03 | ↓ |
| ENSG00000166825 | ANPEP | 0.27 | 0.00 | ↑ | -0.77 | 0.00 | ↓ |
| ENSG00000135315 | CEP162 | 0.27 | 0.00 | ↑ | -0.29 | 0.00 | ↓ |
| ENSG00000275880 | RP11-90L1.8 | 0.27 | 0.04 | ↑ | -0.42 | 0.01 | ↓ |
| ENSG00000141756 | FKBP10 | 0.27 | 0.01 | ↑ | -0.13 | 0.02 | ↓ |
| ENSG00000185504 | FAAP100 | 0.27 | 0.04 | ↑ | -0.19 | 0.03 | ↓ |
| ENSG00000090615 | GOLGA3 | 0.27 | 0.00 | ↑ | -0.17 | 0.01 | ↓ |
| ENSG00000169715 | MT1E | 0.27 | 0.02 | ↑ | -0.60 | 0.04 | ↓ |
| ENSG00000172375 | C2CD2L | 0.27 | 0.00 | ↑ | -0.08 | 0.01 | ↓ |
| ENSG00000047644 | WWC3 | 0.27 | 0.01 | ↑ | -0.16 | 0.03 | ↓ |
| ENSG00000167994 | RAB3IL1 | 0.28 | 0.01 | ↑ | -0.46 | 0.03 | ↓ |
| ENSG00000167110 | GOLGA2 | 0.28 | 0.03 | ↑ | -0.22 | 0.02 | ↓ |
| ENSG00000111077 | TNS2 | 0.28 | 0.00 | ↑ | -0.12 | 0.04 | ↓ |
| ENSG00000112624 | GLTSCR1L | 0.28 | 0.00 | ↑ | -0.12 | 0.03 | ↓ |
| ENSG00000171574 | ZNF584 | 0.28 | 0.02 | ↑ | -0.23 | 0.04 | ↓ |
| ENSG00000228343 | RP11-1148L6.5 | 0.28 | 0.01 | ↑ | -0.20 | 0.00 | ↓ |
| ENSG00000132386 | SERPINF1 | 0.28 | 0.00 | ↑ | -0.10 | 0.02 | ↓ |
| ENSG00000063241 | ISOC2 | 0.28 | 0.00 | ↑ | -0.24 | 0.01 | ↓ |
| ENSG00000140374 | ETFA | 0.28 | 0.01 | ↑ | -0.12 | 0.04 | ↓ |
| ENSG00000174796 | THAP6 | 0.28 | 0.00 | ↑ | -0.18 | 0.03 | ↓ |
| ENSG00000100116 | GCAT | 0.28 | 0.01 | ↑ | -0.12 | 0.05 | ↓ |
| ENSG00000174791 | RIN1 | 0.29 | 0.04 | ↑ | -0.35 | 0.04 | ↓ |
| ENSG00000127311 | HELB | 0.29 | 0.03 | ↑ | -0.19 | 0.03 | ↓ |
| ENSG00000175832 | ETV4 | 0.29 | 0.04 | ↑ | -0.45 | 0.04 | ↓ |
| ENSG00000162227 | TAF6L | 0.29 | 0.00 | ↑ | -0.23 | 0.00 | ↓ |
| ENSG00000171310 | CHST11 | 0.29 | 0.02 | ↑ | -0.27 | 0.01 | ↓ |
| ENSG00000103544 | C16orf62 | 0.29 | 0.00 | ↑ | -0.17 | 0.00 | ↓ |
| ENSG00000186193 | SAPCD2 | 0.29 | 0.00 | ↑ | -0.15 | 0.02 | ↓ |
| ENSG00000251381 | LINC00958 | 0.29 | 0.01 | ↑ | -0.18 | 0.01 | ↓ |
| ENSG00000118193 | KIF14 | 0.30 | 0.00 | ↑ | -0.35 | 0.00 | ↓ |
| ENSG00000140688 | C16orf58 | 0.30 | 0.00 | ↑ | -0.13 | 0.04 | ↓ |
| ENSG00000116771 | AGMAT | 0.30 | 0.02 | ↑ | -0.30 | 0.02 | ↓ |
| ENSG00000013810 | TACC3 | 0.30 | 0.01 | ↑ | -0.17 | 0.04 | ↓ |
| ENSG00000169733 | RFNG | 0.30 | 0.01 | ↑ | -0.17 | 0.01 | ↓ |
| ENSG00000165688 | PMPCA | 0.30 | 0.00 | ↑ | -0.16 | 0.01 | ↓ |
| ENSG00000105355 | PLIN3 | 0.30 | 0.01 | ↑ | -0.12 | 0.04 | ↓ |
| ENSG00000126457 | PRMT1 | 0.30 | 0.01 | ↑ | -0.20 | 0.03 | ↓ |
| ENSG00000162894 | FCMR | 0.31 | 0.00 | ↑ | -0.72 | 0.01 | ↓ |
| ENSG00000119927 | GPAM | 0.31 | 0.01 | ↑ | -0.30 | 0.04 | ↓ |
| ENSG00000134490 | TMEM241 | 0.31 | 0.01 | ↑ | -0.37 | 0.02 | ↓ |
| ENSG00000131591 | C1orf159 | 0.31 | 0.02 | ↑ | -0.17 | 0.04 | ↓ |
| ENSG00000106266 | SNX8 | 0.31 | 0.03 | ↑ | -0.14 | 0.03 | ↓ |
| ENSG00000128944 | KNSTRN | 0.31 | 0.01 | ↑ | -0.23 | 0.05 | ↓ |
| ENSG00000022976 | ZNF839 | 0.32 | 0.05 | ↑ | -0.25 | 0.02 | ↓ |
| ENSG00000130158 | DOCK6 | 0.32 | 0.02 | ↑ | -0.23 | 0.02 | ↓ |
| ENSG00000068028 | RASSF1 | 0.32 | 0.00 | ↑ | -0.14 | 0.01 | ↓ |
| ENSG00000256525 | POLG2 | 0.32 | 0.00 | ↑ | -0.25 | 0.00 | ↓ |
| ENSG00000166851 | PLK1 | 0.32 | 0.00 | ↑ | -0.36 | 0.00 | ↓ |
| ENSG00000171246 | NPTX1 | 0.32 | 0.00 | ↑ | -0.19 | 0.01 | ↓ |
| ENSG00000157617 | C2CD2 | 0.32 | 0.00 | ↑ | -0.27 | 0.00 | ↓ |
| ENSG00000155850 | SLC26A2 | 0.32 | 0.04 | ↑ | -0.39 | 0.04 | ↓ |
| ENSG00000181035 | SLC25A42 | 0.32 | 0.01 | ↑ | -0.08 | 0.05 | ↓ |
| ENSG00000151632 | AKR1C2 | 0.32 | 0.04 | ↑ | -0.28 | 0.04 | ↓ |
| ENSG00000233184 | RP11-421L21.3 | 0.33 | 0.00 | ↑ | -0.36 | 0.03 | ↓ |
| ENSG00000204839 | MROH6 | 0.33 | 0.01 | ↑ | -0.15 | 0.01 | ↓ |
| ENSG00000221955 | SLC12A8 | 0.33 | 0.05 | ↑ | -0.30 | 0.04 | ↓ |
| ENSG00000183691 | NOG | 0.34 | 0.00 | ↑ | -0.19 | 0.01 | ↓ |
| ENSG00000175711 | B3GNTL1 | 0.34 | 0.00 | ↑ | -0.36 | 0.01 | ↓ |
| ENSG00000185262 | UBALD2 | 0.34 | 0.01 | ↑ | -0.13 | 0.02 | ↓ |
| ENSG00000099625 | CBARP | 0.35 | 0.03 | ↑ | -0.24 | 0.02 | ↓ |
| ENSG00000180178 | FAR2P1 | 0.35 | 0.01 | ↑ | -0.36 | 0.01 | ↓ |
| ENSG00000130119 | GNL3L | 0.35 | 0.00 | ↑ | -0.23 | 0.04 | ↓ |
| ENSG00000068489 | PRR11 | 0.35 | 0.00 | ↑ | -0.23 | 0.04 | ↓ |
| ENSG00000141696 | P3H4 | 0.36 | 0.01 | ↑ | -0.03 | 0.00 | ↓ |
| ENSG00000149503 | INCENP | 0.36 | 0.00 | ↑ | -0.18 | 0.02 | ↓ |
| ENSG00000090776 | EFNB1 | 0.36 | 0.01 | ↑ | -0.16 | 0.02 | ↓ |
| ENSG00000178401 | DNAJC22 | 0.37 | 0.02 | ↑ | -0.42 | 0.04 | ↓ |
| ENSG00000179399 | GPC5 | 0.37 | 0.01 | ↑ | -0.44 | 0.00 | ↓ |
| ENSG00000174951 | FUT1 | 0.38 | 0.04 | ↑ | -1.15 | 0.01 | ↓ |
| ENSG00000280385 | AP000648.5 | 0.38 | 0.03 | ↑ | -0.48 | 0.01 | ↓ |
| ENSG00000017483 | SLC38A5 | 0.38 | 0.00 | ↑ | -0.51 | 0.00 | ↓ |
| ENSG00000104522 | TSTA3 | 0.40 | 0.00 | ↑ | -0.27 | 0.02 | ↓ |
| ENSG00000245711 | NADK2-AS1 | 0.40 | 0.01 | ↑ | -0.63 | 0.01 | ↓ |
| ENSG00000109680 | TBC1D19 | 0.40 | 0.04 | ↑ | -0.41 | 0.01 | ↓ |
| ENSG00000280130 | AC073333.1 | 0.41 | 0.05 | ↑ | -0.79 | 0.01 | ↓ |
| ENSG00000128510 | CPA4 | 0.41 | 0.02 | ↑ | -1.08 | 0.00 | ↓ |
| ENSG00000112293 | GPLD1 | 0.41 | 0.01 | ↑ | -0.36 | 0.01 | ↓ |
| ENSG00000124243 | BCAS4 | 0.42 | 0.02 | ↑ | -0.28 | 0.04 | ↓ |
| ENSG00000180953 | ST20 | 0.42 | 0.02 | ↑ | -0.26 | 0.03 | ↓ |
| ENSG00000273576 | RP11-390P24.1 | 0.42 | 0.04 | ↑ | -0.55 | 0.01 | ↓ |
| ENSG00000214654 | RP11-27I1.4 | 0.44 | 0.01 | ↑ | -0.23 | 0.02 | ↓ |
| ENSG00000197355 | UAP1L1 | 0.44 | 0.01 | ↑ | -0.26 | 0.01 | ↓ |
| ENSG00000198074 | AKR1B10 | 0.44 | 0.02 | ↑ | -1.16 | 0.01 | ↓ |
| ENSG00000145860 | RNF145 | 0.44 | 0.00 | ↑ | -0.21 | 0.02 | ↓ |
| ENSG00000153395 | LPCAT1 | 0.45 | 0.00 | ↑ | -0.28 | 0.00 | ↓ |
| ENSG00000269967 | RP11-84A19.4 | 0.45 | 0.03 | ↑ | -1.00 | 0.05 | ↓ |
| ENSG00000254967 | RP11-680F20.6 | 0.47 | 0.02 | ↑ | -0.40 | 0.03 | ↓ |
| ENSG00000219891 | ZSCAN12P1 | 0.47 | 0.03 | ↑ | -0.99 | 0.02 | ↓ |
| ENSG00000173214 | KIAA1919 | 0.47 | 0.00 | ↑ | -0.34 | 0.03 | ↓ |
| ENSG00000125843 | AP5S1 | 0.48 | 0.00 | ↑ | -0.07 | 0.02 | ↓ |
| ENSG00000158552 | ZFAND2B | 0.49 | 0.01 | ↑ | -0.32 | 0.00 | ↓ |
| ENSG00000281404 | LINC01176 | 0.50 | 0.03 | ↑ | -0.43 | 0.01 | ↓ |
| ENSG00000133195 | SLC39A11 | 0.53 | 0.00 | ↑ | -0.33 | 0.01 | ↓ |
| ENSG00000104221 | BRF2 | 0.53 | 0.02 | ↑ | -0.60 | 0.02 | ↓ |
| ENSG00000272525 | RP11-79P5.9 | 0.54 | 0.00 | ↑ | -0.64 | 0.02 | ↓ |
| ENSG00000183963 | SMTN | 0.55 | 0.01 | ↑ | -0.30 | 0.01 | ↓ |
| ENSG00000279602 | CTD-3014M21.1 | 0.55 | 0.04 | ↑ | -0.84 | 0.04 | ↓ |
| ENSG00000236199 | RP11-264I13.2 | 0.55 | 0.04 | ↑ | -0.64 | 0.04 | ↓ |
| ENSG00000213930 | GALT | 0.55 | 0.04 | ↑ | -0.64 | 0.04 | ↓ |
| ENSG00000232713 | AC010733.5 | 0.56 | 0.00 | ↑ | -0.66 | 0.04 | ↓ |
| ENSG00000203362 | RP3-337H4.8 | 0.56 | 0.01 | ↑ | -0.55 | 0.05 | ↓ |
| ENSG00000080823 | MOK | 0.57 | 0.01 | ↑ | -0.31 | 0.03 | ↓ |
| ENSG00000265681 | RPL17 | 0.58 | 0.02 | ↑ | -0.34 | 0.02 | ↓ |
| ENSG00000280355 | RP11-119F19.5 | 0.58 | 0.01 | ↑ | -0.40 | 0.03 | ↓ |
| ENSG00000189171 | S100A13 | 0.58 | 0.04 | ↑ | -0.34 | 0.05 | ↓ |
| ENSG00000269427 | CTC-429P9.1 | 0.59 | 0.00 | ↑ | -0.37 | 0.00 | ↓ |
| ENSG00000251364 | CTD-2516F10.2 | 0.60 | 0.04 | ↑ | -0.50 | 0.02 | ↓ |
| ENSG00000179598 | PLD6 | 0.61 | 0.00 | ↑ | -0.48 | 0.04 | ↓ |
| ENSG00000075886 | TUBA3D | 0.62 | 0.01 | ↑ | -0.39 | 0.02 | ↓ |
| ENSG00000089163 | SIRT4 | 0.62 | 0.01 | ↑ | -0.59 | 0.04 | ↓ |
| ENSG00000232164 | AC092669.3 | 0.65 | 0.01 | ↑ | -0.93 | 0.00 | ↓ |
| ENSG00000185361 | TNFAIP8L1 | 0.66 | 0.00 | ↑ | -0.61 | 0.00 | ↓ |
| ENSG00000265415 | CTD-2510F5.4 | 0.66 | 0.02 | ↑ | -0.45 | 0.03 | ↓ |
| ENSG00000269365 | RP11-380M21.4 | 0.67 | 0.02 | ↑ | -1.57 | 0.01 | ↓ |
| ENSG00000229852 | RP11-398K22.12 | 0.68 | 0.03 | ↑ | -0.43 | 0.04 | ↓ |
| ENSG00000119737 | GPR75 | 0.68 | 0.02 | ↑ | -0.14 | 0.05 | ↓ |
| ENSG00000272763 | RP11-357H14.17 | 0.71 | 0.00 | ↑ | -0.84 | 0.03 | ↓ |
| ENSG00000124614 | RPS10 | 0.73 | 0.02 | ↑ | -0.74 | 0.01 | ↓ |
| ENSG00000279532 | CTB-96E2.6 | 0.74 | 0.02 | ↑ | -0.64 | 0.02 | ↓ |
| ENSG00000279425 | CTD-2017D11.2 | 0.75 | 0.01 | ↑ | -0.53 | 0.00 | ↓ |
| ENSG00000198237 | RP11-98J23.2 | 0.80 | 0.00 | ↑ | -0.40 | 0.00 | ↓ |
| ENSG00000228838 | RP4-784A16.2 | 0.86 | 0.03 | ↑ | -1.59 | 0.03 | ↓ |
| ENSG00000198093 | ZNF649 | 0.87 | 0.04 | ↑ | -0.53 | 0.03 | ↓ |
| ENSG00000259191 | RP11-253M7.3 | 0.88 | 0.01 | ↑ | -1.80 | 0.02 | ↓ |
| ENSG00000244122 | UGT1A7 | 0.90 | 0.01 | ↑ | -0.87 | 0.04 | ↓ |
| ENSG00000253616 | RP11-875O11.3 | 0.91 | 0.01 | ↑ | -1.18 | 0.05 | ↓ |
| ENSG00000110090 | CPT1A | 0.95 | 0.00 | ↑ | -0.20 | 0.00 | ↓ |
| ENSG00000215182 | MUC5AC | 0.96 | 0.02 | ↑ | -0.83 | 0.00 | ↓ |
| ENSG00000271550 | BNIP3P11 | 0.99 | 0.03 | ↑ | -0.94 | 0.02 | ↓ |
| ENSG00000154040 | CABYR | 0.99 | 0.05 | ↑ | -1.17 | 0.02 | ↓ |
| ENSG00000227959 | RP11-276H7.2 | 0.99 | 0.02 | ↑ | -0.90 | 0.02 | ↓ |
| ENSG00000205090 | TMEM240 | 1.03 | 0.04 | ↑ | -0.32 | 0.01 | ↓ |
| ENSG00000273759 | RP4-563E14.1 | 1.17 | 0.03 | ↑ | -0.70 | 0.03 | ↓ |
| ENSG00000204428 | LY6G5C | 1.18 | 0.04 | ↑ | -1.09 | 0.01 | ↓ |
| ENSG00000236305 | RP11-126L15.4 | 1.20 | 0.00 | ↑ | -0.68 | 0.00 | ↓ |
| ENSG00000232762 | RP4-784A16.4 | 1.27 | 0.01 | ↑ | -1.40 | 0.02 | ↓ |
| ENSG00000274849 | RP11-49I11.4 | 1.30 | 0.01 | ↑ | -0.72 | 0.03 | ↓ |
| ENSG00000230185 | C9orf147 | 1.31 | 0.02 | ↑ | -0.92 | 0.03 | ↓ |
| ENSG00000166333 | ILK | 1.44 | 0.01 | ↑ | -0.86 | 0.00 | ↓ |
| ENSG00000262372 | RP11-669E14.6 | 1.50 | 0.01 | ↑ | -1.20 | 0.05 | ↓ |
| ENSG00000230415 | RP5-902P8.10 | 1.59 | 0.01 | ↑ | -0.72 | 0.04 | ↓ |
| ENSG00000273338 | RP11-386I14.4 | 1.59 | 0.01 | ↑ | -1.48 | 0.04 | ↓ |
| ENSG00000267284 | RP11-397A16.1 | 1.65 | 0.01 | ↑ | -0.56 | 0.01 | ↓ |
| ENSG00000268756 | AC104534.2 | 1.66 | 0.05 | ↑ | -0.84 | 0.01 | ↓ |
| ENSG00000225447 | RPS15AP10 | 1.66 | 0.01 | ↑ | -1.56 | 0.01 | ↓ |
| ENSG00000276292 | RP11-33N14.5 | 1.69 | 0.03 | ↑ | -1.26 | 0.01 | ↓ |
| ENSG00000279203 | AC005785.5 | 1.95 | 0.01 | ↑ | -0.97 | 0.03 | ↓ |
| ENSG00000279599 | AC005514.2 | 2.12 | 0.04 | ↑ | -2.02 | 0.05 | ↓ |
| ENSG00000248256 | OCIAD1-AS1 | 2.36 | 0.04 | ↑ | -1.29 | 0.02 | ↓ |
| ENSG00000272279 | RP11-157J24.2 | 2.54 | 0.00 | ↑ | -0.63 | 0.04 | ↓ |
| ENSG00000275401 | RP4-564F22.7 | 2.68 | 0.01 | ↑ | -0.84 | 0.01 | ↓ |
| ENSG00000231073 | RP11-316M1.3 | 3.11 | 0.01 | ↑ | -0.52 | 0.03 | ↓ |
| ENSG00000228634 | RP4-534N18.2 | 3.75 | 0.00 | ↑ | -1.09 | 0.04 | ↓ |
| ENSG00000231201 | AF127577.11 | 3.75 | 0.00 | ↑ | -1.11 | 0.03 | ↓ |

**Table: The list of differential expressed genes between Model vs Control and AKG vs Model**
